# One pot synthesis of two potent Ag(I) complexes with quinoxaline ligand, X-ray structure, Hirshfeld analysis, antimicrobial, and antitumor investigations

**DOI:** 10.1038/s41598-022-24030-x

**Published:** 2022-12-03

**Authors:** Mostafa A. El-Naggar, Mona Mohammed Sharaf, Jörg H. Albering, Morsy A. M. Abu-Youssef, Taher S. Kassem, Saied M. Soliman, Ahmed M. A. Badr

**Affiliations:** 1 Department of Chemistry, Faculty of Science, Alexandria University, P.O. Box 426, Ibrahimia, 21321 Alexandria Egypt; 2 Protein Research Department, Genetic Engineering and Biotechnology Research Institute, City of Scientific Research and Technological Applications, Alexandria, Egypt; 3 Graz University of Technology, Mandellstr. 11/III, 8010 Graz, Austria

**Keywords:** Chemical biology, Chemistry

## Abstract

In one pot, the self-assembly of AgNO_3_ and 2-chloroquinoxaline (**2Cl-quinox**) in water–ethanol mixture afforded two novel crystalline Ag(I) complexes. The major product is the polymeric complex **[Ag(2Cl-quinox)(NO**_**3**_**)]**_**n**_; **(1)**, while the minor product (**2**) comprises two molecules which are the monomeric **[Ag(2Cl-quinox)**_**2**_**(NO**_**3**_**)]**; (**2a)** and polymeric **[Ag(2Cl-quinox)(NO**_**3**_**)]**_**n**_; **(2b)** complexes. The single crystal X-ray structure revealed that **1** and **2b** are made up of two-dimensional infinite sheets. In contrast, **2a** is a monomeric complex which has a highly distorted tetrahedral geometry around Ag(I) center. In all cases, the **2Cl-quinox** molecule acts as a terminal monodentate ligand. Complexes **1** and **2b** have similar molecular structures and also have almost similar crystal packing. Using Hirshfeld surface analysis, the O…H hydrogen bonds and π–π stacking interactions contributed significantly to the molecular packing. Both complexes have broad-spectrum action towards multi drug-resistance bacteria. The most effective function of **2** is against *Proteus morganii*, with a MIC value of 8 μg/mL. Complex **2** (IC_50_ = 5.93 ± 0.52 μg/mL) has remarkably greater cytotoxic effect against lung carcinoma (A-549) than *cis*-platin (IC_50_ = 7.5 ± 0.69 μg/mL) and AgNO_3_ (IC_50_ = 14.7 ± 0.53 μg/mL). The higher Ag-content in **2** could be the main reason for its higher cytotoxicity than **1**.

## Introduction

Microbial drug resistance motivates researchers to look for new materials to treat these harmful pathogenic microbes^1^. This search is particularly necessary in the light of the growing number of antibiotic-resistant strains and the greater challenges associated with infection treatment. Silver has been utilized as an antibacterial agent, whether in its metallic form or as different Ag(I) compounds^2–6^. Despite the fact that *N*-heterocyclic ligands demonstrate diverse biological actions against various pathogens^7–10^, their activity is enhanced when they are coordinated with Ag(I) ion^11,12^. The constituents of the silver(I) complexes are likely to work together in a synergistic manner. The significant antimicrobial activities of silver(I) complexes containing *N*-heterocycles were approved in the literature^13–23^. The antimicrobial properties of silver complexes are expected to be influenced primarily by the nature of the atoms coordinated to the silver center and the ease of ligand replacement. Silver(I) complexes with a higher likelihood of ligand replacement by biological ligands (sulfur-containing molecules) are powerful antimicrobial agents. Therefore, it makes sense that complexes with weak metal–ligand bonds, such as those with Ag–N and Ag–O, would exhibit a broader range of antimicrobial activity than those with Ag–S and Ag–P bonds whose silver complexes have weak or no activity against bacteria, molds, and yeast^24–27^. Furthermore, complexes of Ag(I) have attracted attention for cancer treatments in recent years. A set of review articles emphasized on Ag(I) complexes with *N*-heterocycles has targeted their uses as anticancer agents against a variety of cancer cell lines^28^. It has been found that several Ag(I) complexes have greater cytotoxicity than *cis*-platin with a relatively little poisonous effects to the healthy human cells^29–36^.

**Figure 1:**
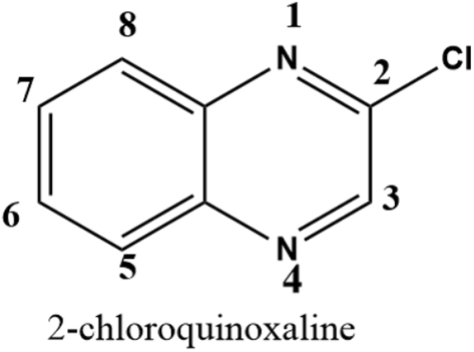
Structure of 2-chloroquinoxaline (**2Cl-quinox**).

Quinoxaline derivatives have been established to have antifungal, antibacterial, and anticancer activities^37–46^. The combination of quinoxaline with Ag(I) ions results in the formation of complexes with fascinating biological characteristics, which could be an interesting alternatives to the current medications^47^. The current aim is to create a new synergic drug by combining bio-active metal ion such as Ag(I) with an effective organic ligand such as 2-chloroquinoxaline (Fig. 1). The syntheses, as well as the spectral and structural analyses of the target complexes were performed using single crystal X-ray diffraction, FTIR and ^1^H NMR spectroscopic methods. Furthermore, determining the antimicrobial potential of the complexes as zone of inhibition and MIC (minimum inhibitory concentration) allowed us to compare their action to that of commercially available antibiotics. Also, the anticancer activity of the novel silver(I) complexes is discussed.

## Materials and methods

### Chemicals and physicochemical characterizations

All of the chemicals were obtained from Sigma-Aldrich Company. The FTIR spectra were achieved at 4000–400 cm^−1^ using a Bruker Tensor 37 FTIR instrument in KBr pellets. The ^1^H NMR spectra of the studied complexes and the free ligand were collected on a JEOL JNM-ECA 500 MHz NMR spectrometer in DMSO-*d*_*6*_ as solvent. Perkin Elmer 2400 Elemental Analyzer was used for the CHN analysis. The amount of Ag content was measured using a Shimadzu atomic absorption spectrophotometer (AA-7000 series, Shimadzu, Ltd., Japan). Thermogravimetric analysis was carried out using a LINSEIS STA PT100 thermogravimetric analyzer in a platinum cell with a heating rate of 10 °C/min and under a nitrogen flow of 40 mL/min.

### Syntheses of [Ag(2Cl-quinox)(NO_3_)]; (1) and [Ag_3_(2Cl-quinox)_4_(NO_3_)_3_]; (2)

**Figure 2:**
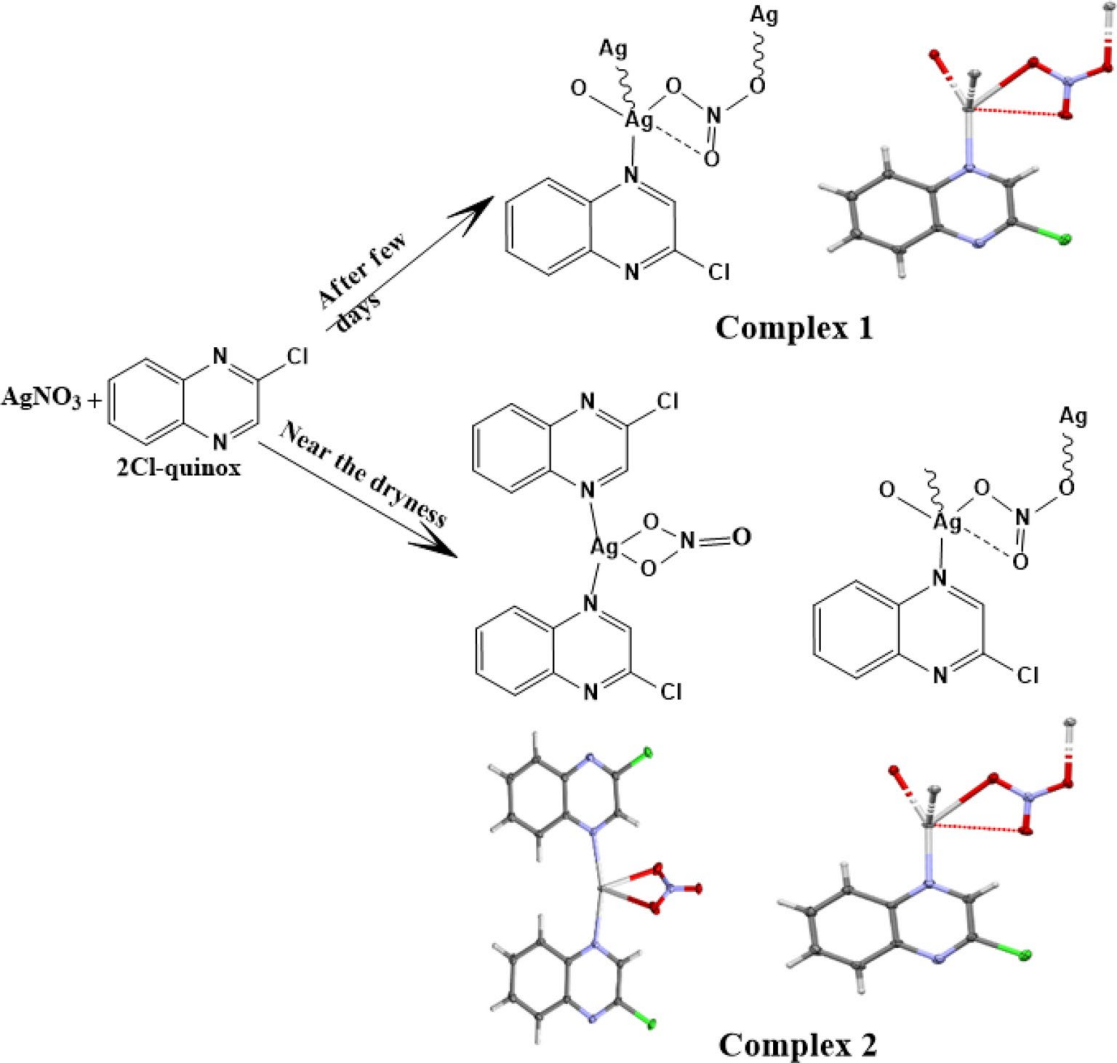
Scheme of the Synthesis of complexes **1** and **2**.

At room temperature, an ethanolic solution (10 mL) of **2Cl-quinox** (165 mg, 1 mmol) was added to a 10 mL aqueous solution of silver(I) nitrate (170 mg, 1 mmol). This mixture was left at room temperature and allowed to evaporate slowly. Complex **1** was obtained as colorless plate crystals after a few days. Near the dryness of the solution, complex **2** is obtained as needle crystals. The obtained crystals were found suitable for X-ray single crystal measurements. Schematic presentation for the synthesis of complexes **1** and **2** is shown in Fig. 2.

**[Ag(2Cl-quinox)(NO**_**3**_**)]**_**n**_; (**1**) (74% yield). Anal. Calc. C_8_H_5_AgClN_3_O_3_: C, 28.73; H, 1.51; N, 12.56; Ag, 32.25%. Found: C, 28.92; H, 1.46; N, 12.40; Ag, 32.01%. FTIR cm^−1^: 3519, 3446, 3056, 2395, 1540, 1489, 1383, 1155, 1093, 960, 760, 591, 454, 415. **2Cl-quinox**: 3388, 3041, 2894, 1675, 1610, 1558, 1518, 1396, 1309, 1182, 763, 625, 580, 520, 466. (Figs. S1–S2, Supplementary data). ^1^H NMR (500 MHz, DMSO-*d*_*6*_) δ_H_ (ppm): 8.99 (s, 1H, C(3)H), 8.12 (d, 1H, C(8)H), 8.03 (d, 1H, C(5)H), 7.89 (t, 2H, C(6)H and C(7)H). Ligand (**2Cl-quinox**): 8.97 (s, 1H, C(3)H), 8.12 (d, 1H, C(8)H), 8.03 (d, 1H, C(5)H), 7.89 (t, 2H, C(6)H and C(7)H) (Figs. S3–S4, Supplementary data).

**[Ag**_**3**_**(2Cl-quinox)**_**4**_**(NO**_**3**_**)**_**3**_**]**; (**2**) Anal. Calc. C_32_H_20_Ag_3_Cl_4_N_11_O_9_: C, 32.91; H, 1.73; N, 13.19; Ag, 27.71%. Found: C, 32.76; H, 1.68; N, 13.02; Ag, 27.53%. FTIR cm^−1^: 3059, 2400, 1687, 1540, 1489, 1383, 1304, 1249, 1156, 1095, 961, 761, 591, 457, 416. (Fig. S5, Supplementary data). ^1^H NMR (500 MHz, DMSO-*d*_*6*_) δ_H_ (ppm): 8.94 (s, 3H, C(3)H), 8.12 (d, 3H, C(8)H), 8.03 (d, 3H, C(5)H), 7.90 (t, 6H, C(6)H and C(7)H). (Fig. S6, Supplementary data).

### Crystal structure analysis

A Bruker APEX II diffractometer with graphite monochromated MoKα radiation was used to determine the crystal structures of the investigated complexes. Table S1 (Supplementary data) contains all information pertaining to the crystallographic measurements. SADABS was used for absorption corrections^48^ and all calculations were carried out using SHELXTL program package^49^.

### Hirshfeld surface analysis

The Crystal Explorer Ver. 3.1^50^ program package was used to analyze the Hirshfeld surface and draw the 2D fingerprint plots.

### Biological studies

#### Determination of antimicrobial and cytotoxic activities

The antimicrobial evaluation of the Ag(I) complexes towards Gram-positive bacteria, Gram-negative bacteria, and harmful yeasts was determined^11^. More details are found in Method S1 (Supplementary data). The cytotoxic activity of the Ag(I) complexes against the lung carcinoma (A-549) and breast carcinoma (MCF-7) cell lines was determined using the procedure described in Method S2 (Supplementary data)^51^.

## Results and discussion

### X-ray crystal structure descriptions

#### Crystal structure of [Ag(2Cl-quinox)(NO_3_)]_n_; (1)

**Figure 3:**
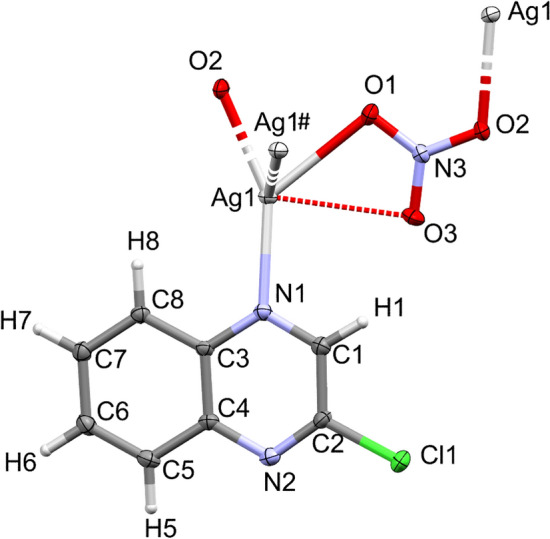
Displacement ellipsoids drawing (50% probability) of **1**, showing the atom-numbering scheme. The bonds, Ag1-O2 and Ag1–Ag1#, are shown with a broken line, because the O2 and Ag1# atoms belong to the neighboring monomer units.

The complex **[Ag(2Cl-quinox)(NO**_**3**_**)]**_**n**_ crystallizes in the monoclinic space group *C2/c* with Z = 8 and one **[Ag(2Cl-quinox)(NO**_**3**_**)]** monomeric unit as an asymmetric formula. Figure 3 shows the coordination geometry and the atom numbering scheme of this complex. The monomeric unit of this two-dimensional infinite coordination polymer includes one organic ligand per AgNO_3_. Selected interatomic distances and bond angles are presented in Table S2 (Supplementary data). The geometry around the Ag(I) ion in complex **1** could be considered as a distorted triangular planar augmented by strong argentophilic interaction with the symmetry related Ag1# atom (Symm. code: # 1/2 − x, 1/2 − y, 2 − z) which forms the tip of a triangular pyramid. The base of this triangular pyramid is constructed by the atoms: O1 and O2# (Symm. code: # 1/2 − x, 1/2 + y, 2.5 − z) of the nitrate anions and N1 of the quinoxaline ligand. The nitrate anion acts as a bridging ligand between the Ag(I) sites via Ag(1)–O(1) and Ag(1)–O(2) bonds with silver to oxygen distances of 2.5502(15) and 2.2602(14) Å, respectively. An additional weak Ag1…O3 interaction has a distance of 2.829 Å which is significantly long and could not be considered as a bond. Hence, the coordination number of the Ag(I) is 3.

**Figure 4:**
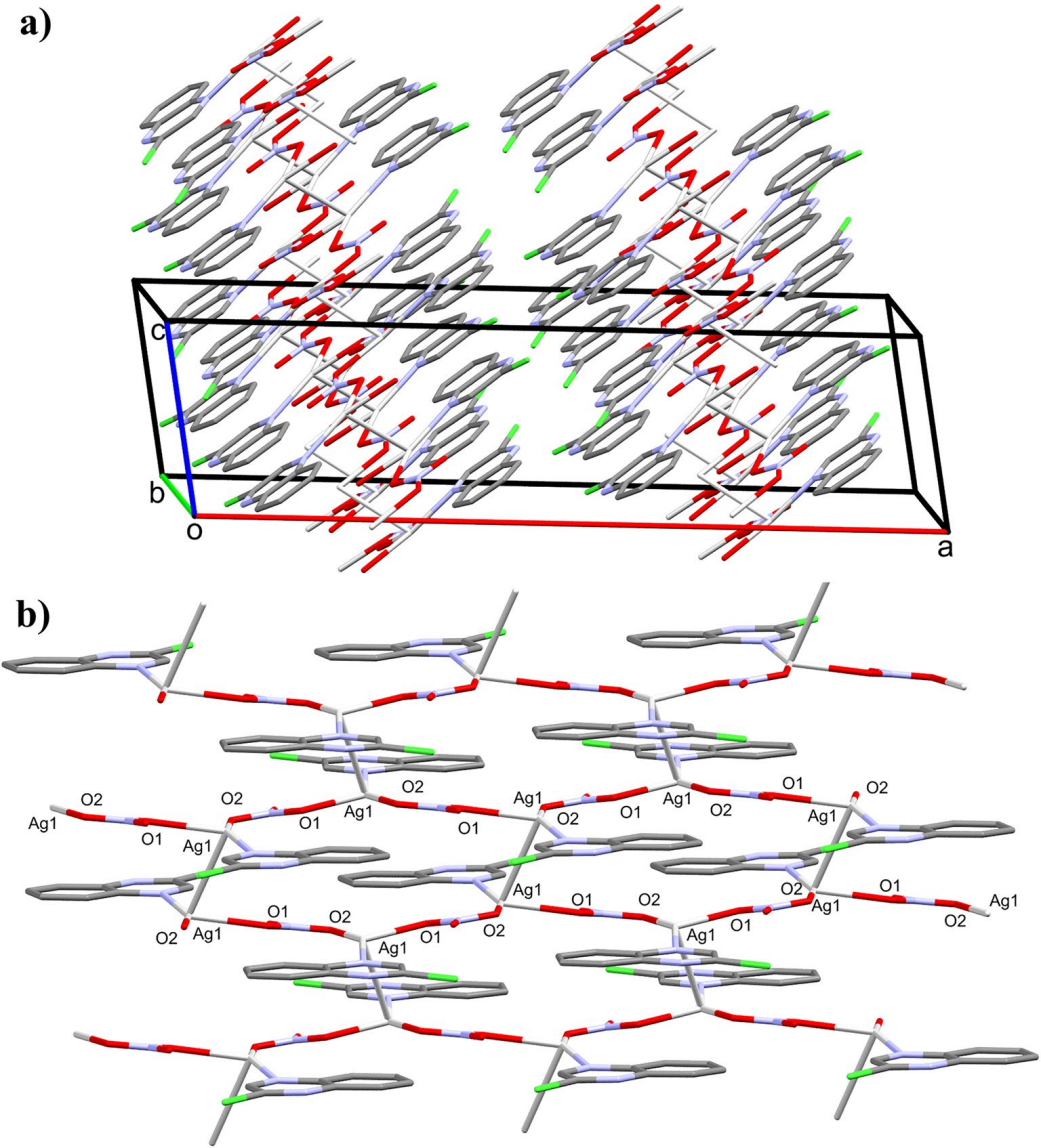
Projections of the crystal packing in the unit cell (**a**), and the two-dimensional infinite coordination polymer of complex **1** (**b**). All hydrogen atoms are omitted from this figure for better visualization.

As shown in Fig. 4, the crystal structure of **1** is made up of two-dimensional infinite sheets that expand perpendicular to the *a*-direction of the unit cell via strong Ag–O and Ag–Ag bonds. The alternating silver Ag1 and the oxygen atoms O1 and O2 of the bridging nitrate groups are connected in a wavy-like and coplanar pattern along the crystallographic *b*-direction, while the organic ligand units are arranged in a highly symmetric manner below and above the polymer array. These parallel polymeric chains are cross-linked by the argentophillic interactions along the *c*-direction in the unit cell where the Ag1−Ag1# (Symm.: #1/2 − x, 1/2 − y, 2 − z) bond distance is 3.1008(4) Å. This distance is significantly short, indicating significant argentophillic interactions^52^ which are responsible for the extension of the 2D polymer along the *c*-direction. As we note in the two units attached by the argentophilic interaction, the two quinoxaline ligands have an anti-configuration to one another.

**Figure 5:**
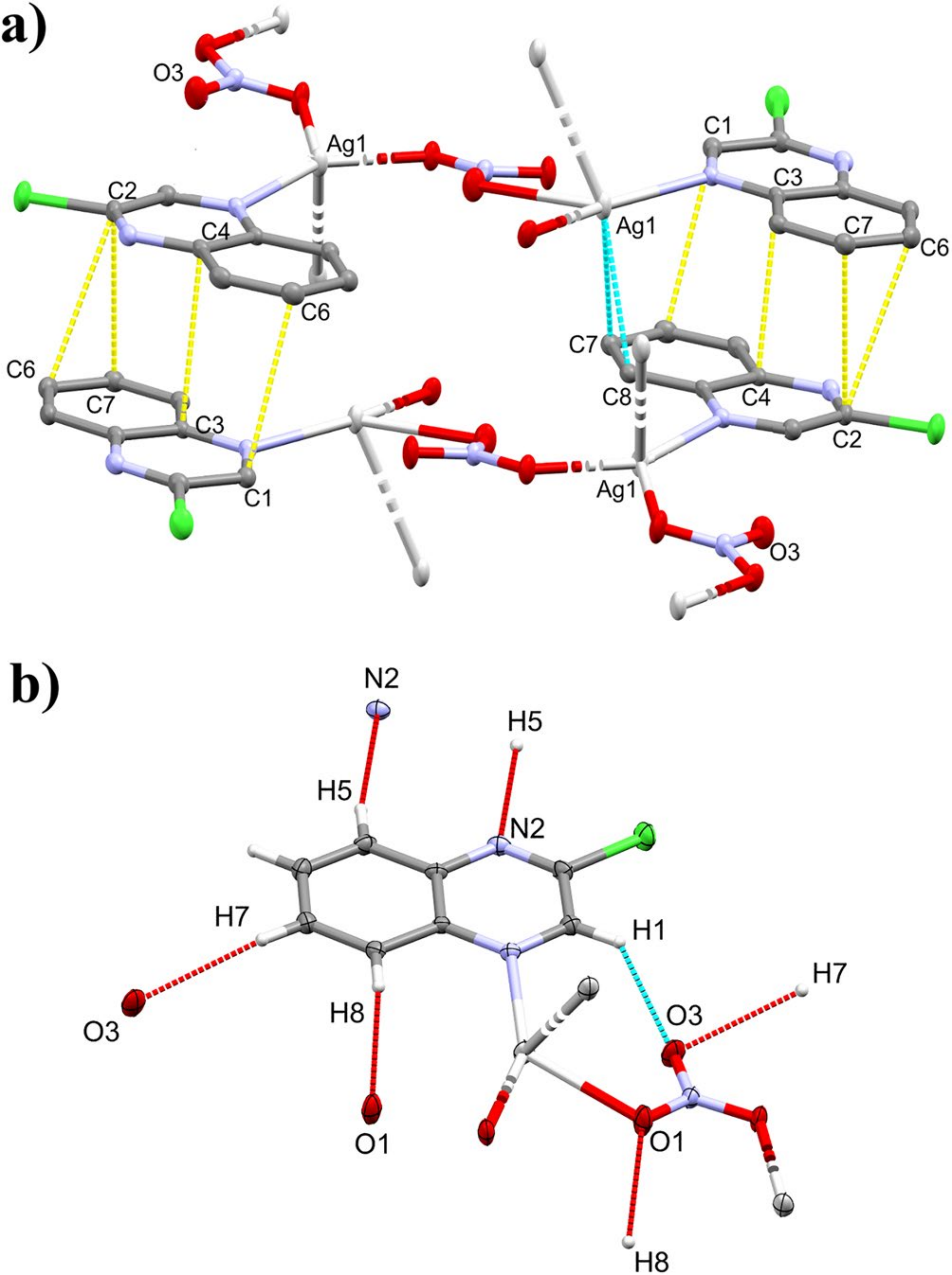
Crystal packing of **1** via C…C (yellow), Ag…C (light blue) interactions (**a**) and hydrogen bond contacts (**b**).

In addition to the argentophilic interactions which play significant role in the polymer expansion along *c*-direction, there are many π–π stacking interactions between the quinoxaline rings which are found approximately parallel to each other. The shortest C…C contacts are C1…C6 (3.395 Å), C3…C4 (3.299 Å), C2…C6 (3.391 Å) and C2…C7 (3.242 Å) interactions. For better clarity, these π-π stacking interactions are displayed as yellow dotted lines in Fig. 5. Moreover, the distance between the ring centroids C3C4C5C6C7C8 and C1C2C3C4N1N2 is 3.528 Å which agrees with the reported values for the π–π stacking interactions in the aromatic six membered ring system^53^. Also, there are additional Ag…C interactions such as Ag1…C7 and Ag1…C8 contacts (Fig. 5). The corresponding interaction distances are 2.945 and 3.029 Å, respectively. Besides these interactions, the complex units are packed together via the hydrogen bridge bonds such as O…H (along the *b*-axis) and N…H (along the *a*-axis) contacts as given in Table S3 (Supplementary data) and displayed in Fig. 5.

#### Crystal structure of [Ag_3_(2Cl-quinox)_4_(NO_3_)_3_]; (2)

**Figure 6:**
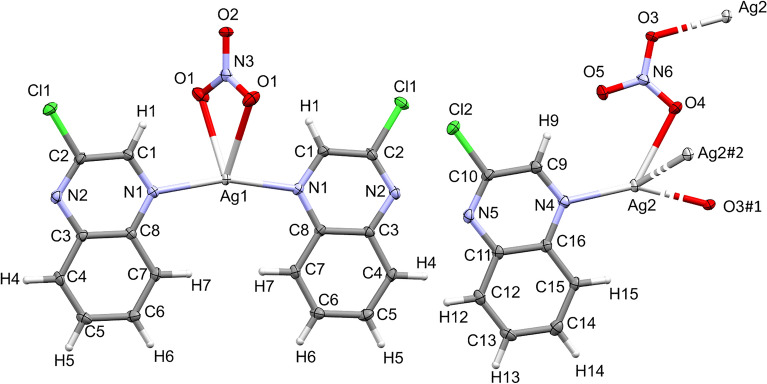
Displacement ellipsoids drawing (50% probability) of **2**, showing the atom-numbering scheme. It consists of two complex units, **2a** (left) and **2b** (right). The Ag2–O3#1 and Ag2–Ag2#2 bonds in **2b**, are shown with a broken line, because the O3#1 and Ag2#2 atoms belong to the neighboring monomer units, but still belong to the coordination sphere of the silver atom. Symmetry codes to generate O3#1 and Ag2#2 are 1/2 − x, − 1/2 + y, − 1/2 − z and 1/2 − z, 1/2 − y, − z, respectively.

The crystal structure of complex **2** is presented in Fig. 6 while the crystal refinement data is listed in Table S1, Supplementary data. Also, it crystallizes in the monoclinic centrosymmetric space group *C2/c*. The parameters of the unit cell are *a* = 50.235(2) Å, *b* = 9.7041(4) Å, *c* = 7.3220(3) Å and *β* = 91.536(2)° while the unit cell volume is 3568.1(3) Å^3^. It consists of two different complex molecules: **[Ag(2Cl-quinox)**_**2**_**(NO**_**3**_**)]; (2a)** and **[Ag(2Cl-quinox)(NO**_**3**_**)]**_**n**_; (**2b)** which represent the monomeric and polymeric parts of this complex, respectively. The crystal structure of complex **2b** is almost the same as that for complex **1**, so the discussion of complex **2** will focus only on the monomer **2a**. In this monomeric complex , the Ag(I) is coordinated with two **2Cl-quinox** ligand units through the heterocyclic *N*-atom where the two ligand units are in *syn* configuration to one another, and two oxygen atoms belonging to a bidentate nitrate group (O1 and O1#, Symmetry code #: − x, y, 1/2 − z) indicating a highly distorted tetrahedral geometry around Ag(I) center. Because of the symmetry consideration, the two Ag–N bonds are equidistant (2.243(2) Å). The same is true for the two Ag–O bonds (2.613(2) Å) (Table S4**,** Supplementary data). It is clear that the two coordinating quinoxaline systems are nearly co-planar, where the angle between their ring planes is only 3.7° (Fig. S7, Supplementary data). Also, the mean plane passing through the two quinoxaline moieties adopts an angle of 61.31^◦^ to the plane constructed from the bidentate nitrate group (O1, O1#, O2, N3).

**Figure 7:**
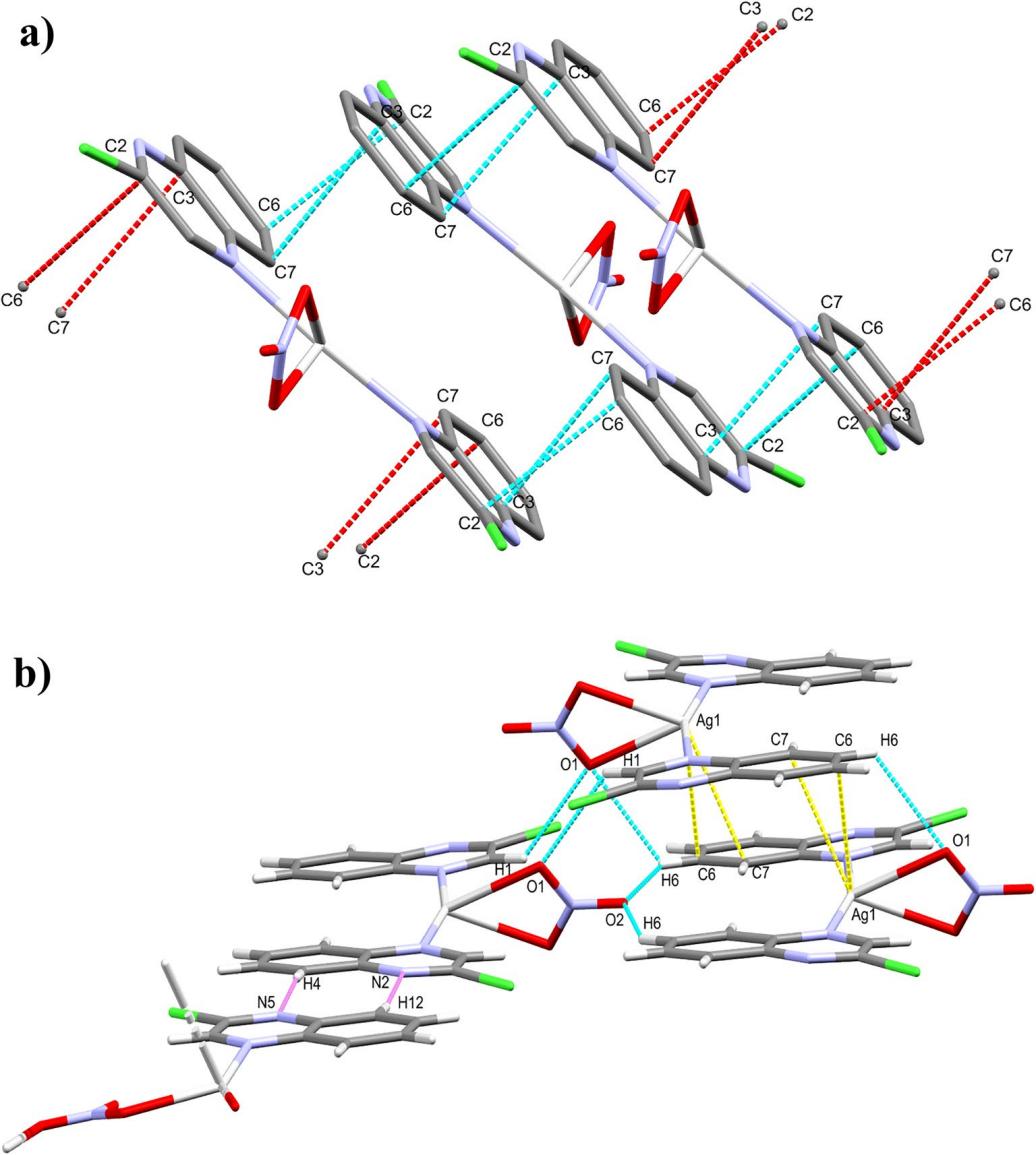
The π–π stacking interactions in complex **2a** (**a**). The Crystal packing of **2** via O…H (light blue), Ag…C (yellow) and N…H (violet) interactions (**b**).

Also, the packing of complex **2** include some π–π stacking contacts between the aromatic ring π-systems of **2a** where the C2…C6 (3.231 Å) and C3…C7 (3.386 Å) are the most important π-π interactions. Also, the centroid–centroid distances are found to be in the range 3.647–3.851 Ǻ, which are generally larger than those found in **1** (Fig. 7). The **2a** units in complex **2** are also packed together via Ag1…C6 (3.172 Å) and Ag1…C7 (3.236 A˚) intermolecular interactions as well as the O…H hydrogen bridge bonds. Details regarding the hydrogen bond parameters are given in Table S5 (Supplementary data). In addition, the two complexes **2a** and **2b** are interconnected in the crystal structure of complex **2** via N2…H12 (2.708 Å) and N5…H4 (2.503 Å) hydrogen bonds. For better clarity, these interactions are displayed as violet dotted lines in Fig. 7.

**Figure 8:**
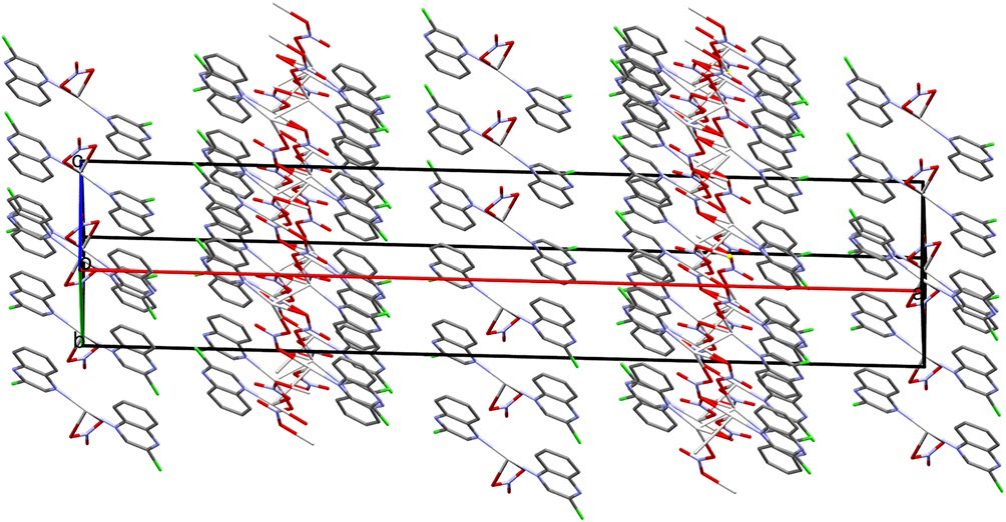
Projections of the molecular packing of **2** in the unit cell where the 2D polymeric units **2a** and the monomeric complex **2b** are arranged alternatively. All hydrogen atoms are left out from this figure for better visualization.

In comparison between complexes **1** and **2**, the molecular packing of the former is simply described as a 2D coordination polymer via the nitrate ion as connector between Ag(I) sites which are further connected by Ag–Ag bonds (Fig. 4). On the other hand, the molecular packing of **2** is built from the alternative arrangement of the monomeric complex **2a** and 2D polymer chain **2b** (Fig. 8).

Unlike complexes **1** and **2**, the previously reported X-ray structure of the structurally related [Ag(quinoxaline)]_n_(NO_3_)_n_ complex revealed its polymeric nature through the two nitrogen atoms of quinoxaline as a connector ligand between the Ag(I) sites^47^. Generally, this bridging mode of the quinoxaline ligands is usual in its complexes^54,55^. Unexpectedly, the 2-chloroquinoxaline in the studied complexes acts as a terminal monodentate ligand rather than a connector where the coordination with Ag(I) occurs only through one nitrogen atom while the other nitrogen does not participate in coordination with Ag(I) as a consequence of the decrease in its electron density due to its proximity to the electron attracting group (chlorine atom). In contrast, the freely uncoordinated N-atoms contribute significantly to the molecular packing by connecting the parts **2a** and **2b** via N…H hydrogen bonding interactions.

### FTIR spectra

The infrared spectra of complexes **1** and **2** compared to the ligand, **2Cl-quinox**, have been measured and are shown in Figs. S1, S2 and S5 (Supplementary data). The IR spectrum of complex **1** is very similar to that of complex **2.** The bands appeared in the range of 3041–3059 cm^−1^ in the FTIR spectra of the complexes, and their free ligand could be assigned to the ν(C–H) aromatic stretching vibrations. The bands appear at 1610 cm^−1^ for the ligand and 1540 cm^−1^ for both complexes can be attributed to the stretching vibrational frequency of the C=N bond. As we note, the observable shift of ν(C=N) band to a lower frequency in the spectra of both complexes indicates its involvement in the coordination to the silver metal. In the FTIR spectra of complexes **1** and **2**, the presence of a sharp spectral band at 1383 cm^−1^ indicates the presence of the nitrate group, which doesn’t exist in the FTIR spectrum of the free ligand. Furthermore, in the regions of 591 and 414–457 cm^−1^, some new moderate-intensity bands are detected in the complexes spectra. These bands might be attributed to the vibrational modes of the Ag–O and Ag–N bonds, respectively^56^.

### NMR spectra

The ^1^H NMR spectra of **2Cl-quinox** compared to the complexes **1** and **2** are presented in Figs. S3, S4 and S6 (Supplementary data) while the chemical shifts and their assignments are summarized in Table S6 (Supplementary data) which shows no important shifts upon complexation of the free ligand. This is generally known for silver (I) complexes with N-donor ligands, which have relatively weak Ag–N and Ag–O interactions^57^. The spectral data is consistent with the structure of the organic part of complexes **1** and **2**. The ^1^H NMR spectra of **2Cl-quinox** and the corresponding Ag(I) complexes give four signals associated to the aromatic protons. Two doublet signals for the protons C(5)H and C(8)H, one triplet signal for C(6)H and C(7)H, and one singlet peak for C(3)H. The chemical shift values decrease in order C(3)H > C(8)H > C(5)H > C(6)H and C(7)H (Table S6, Supplementary data). The singlet peak for C(3)H has the largest chemical shift value as it is adjacent to the chlorine and nitrogen atoms. These NMR spectra give a good indication on the purity of the studied complexes.

### Thermal analysis

Thermogravimetric (TG) and differential thermal analyses (DTA) curves for complexes **1** and **2** are displayed in Fig. S8 (Supplementary data), while the summary of their thermal decomposition is listed in Table S7 (Supplementary data). The TGA and DTA curves revealed that complexes **1** and** 2** are thermally decomposed in two successive decomposition steps. The first one is an endothermic step, while the second one is exothermic. For **1**, the first step represents the removal of the ligand with an estimated loss of 49.639% (theoretical loss = 49.211%) up to a temperature of 220 °C. The second step occurred within the temperature range 220–600 °C with an estimated loss of 18.175% (theoretical loss of about 18.539%), which is reasonably accounted for the successive liberation of [NO_2_ + 0.5O_2_], leaving a residue of metallic silver [Ag] of about 32.186% (theoretical mass about 32.251%). On the other hand, four molecules of **2Cl-quinox** are lost in the first step of the thermal decomposition of complex **2** up to 240 °C, with an estimated loss of 55.423% (theoretical loss of about 56.367%). The second step started at a temperature of 240 °C, leaving a residue of 27.939% for the metallic silver with a theoretical mass percent 27.706% at the end of the thermal decomposition (800 °C) with an estimated loss of 15.638% (theoretical loss = 15.927%) which is reasonably accounted for the successive liberation of 3[NO_2_ + 0.5O_2_].

### Analysis of Hirshfeld surface

**Figure 9:**
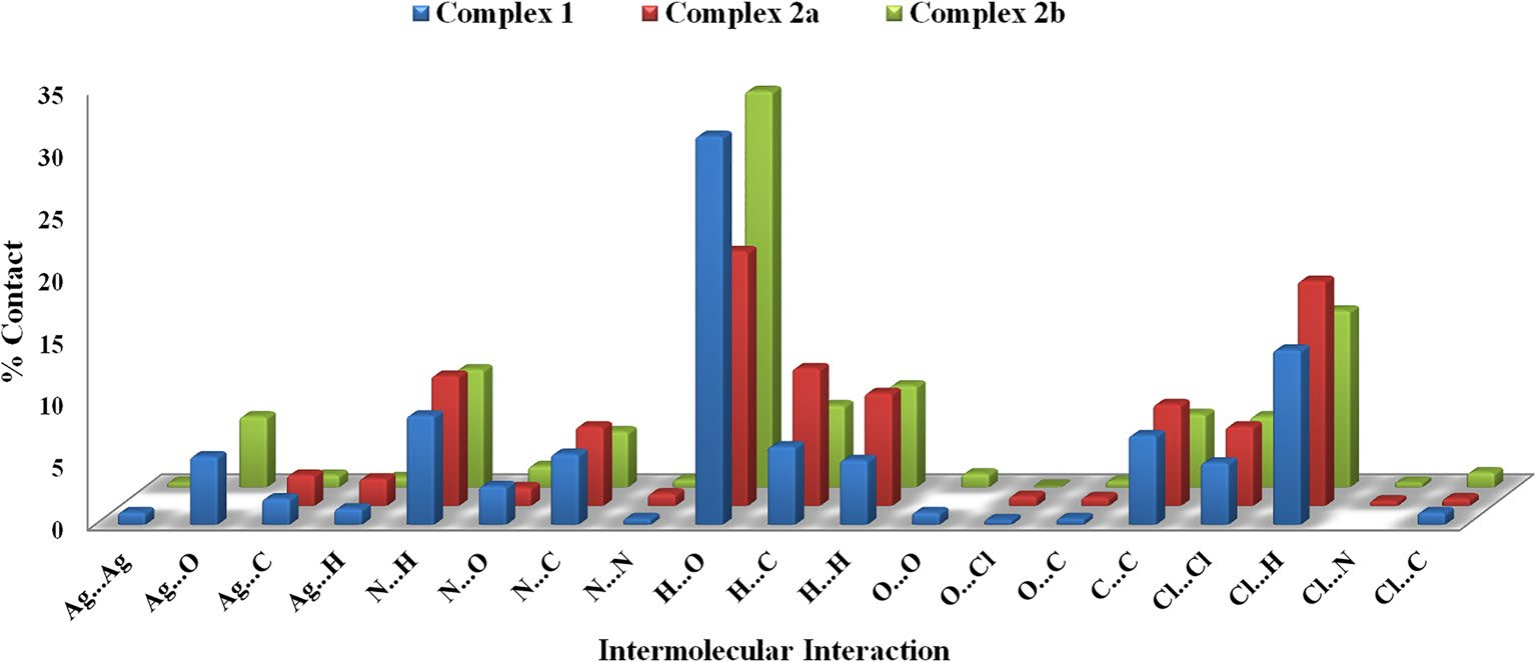
The percentage contributions of all intermolecular interactions in the crystal structure of the investigated Ag(I) complexes.

Qualitative and quantitative analyses of the intermolecular contacts in the investigated Ag(I) complexes were carried out utilizing Hirshfeld surface analysis. In contrast to **1**, the asymmetric unit of complex **2** consists of two different complex molecules, **2a** and **2b**, which represent the monomeric and polymeric parts of this complex, respectively (Fig. 6). Hence, the Hirshfeld surfaces of each molecule are calculated separately. The contributions of all intermolecular contacts to the total fingerprint plots of the crystalline structure of complexes **1**, **2a**, and **2b** are depicted in **Fig. ****9**. Hirshfeld analysis illuminated the importance of the O…H, H…Cl, N…H, C…H, H…H, C…C and C…N interactions on the packing of the crystal structure of the investigated compounds.

**Figure 10:**
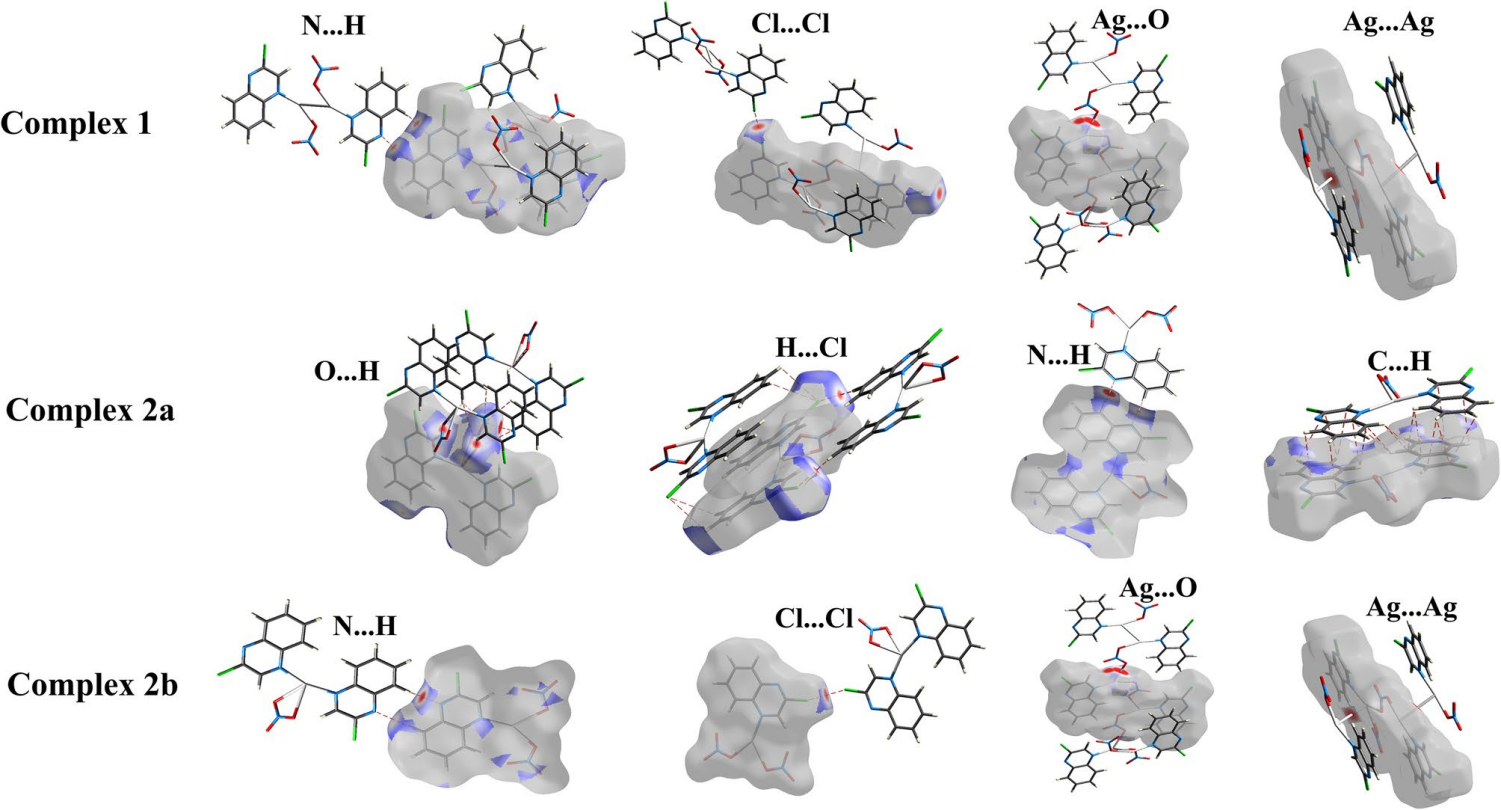
The d_norm_ maps of the most significant contacts in compounds **1** and **2**.

The d_norm_ maps for the most important contacts in the complexes under investigation are shown in Fig. 10. Interestingly, complexes **1** and **2b** have slightly different packing and almost the same interaction patterns. The most significant interactions in **1** and **2b**, which have almost similar structure, are Ag–O, Ag–Ag, N…H and Cl…Cl. In both cases, the Ag–O and Ag–Ag contacts occurred within the polymeric part of **1** and **2b.** In contrast, the N…H and Cl…Cl interactions in complex **1** are different from those in **2b**. In the former, the N…H and Cl…Cl interactions occur among the 2D polymer chains. The situation is totally different in complex **2**, where the N…H and Cl…Cl interactions occur between the 2D polymer chains of **2b** and monomeric units of **2a** (Fig. 10).

**Figure 11:**
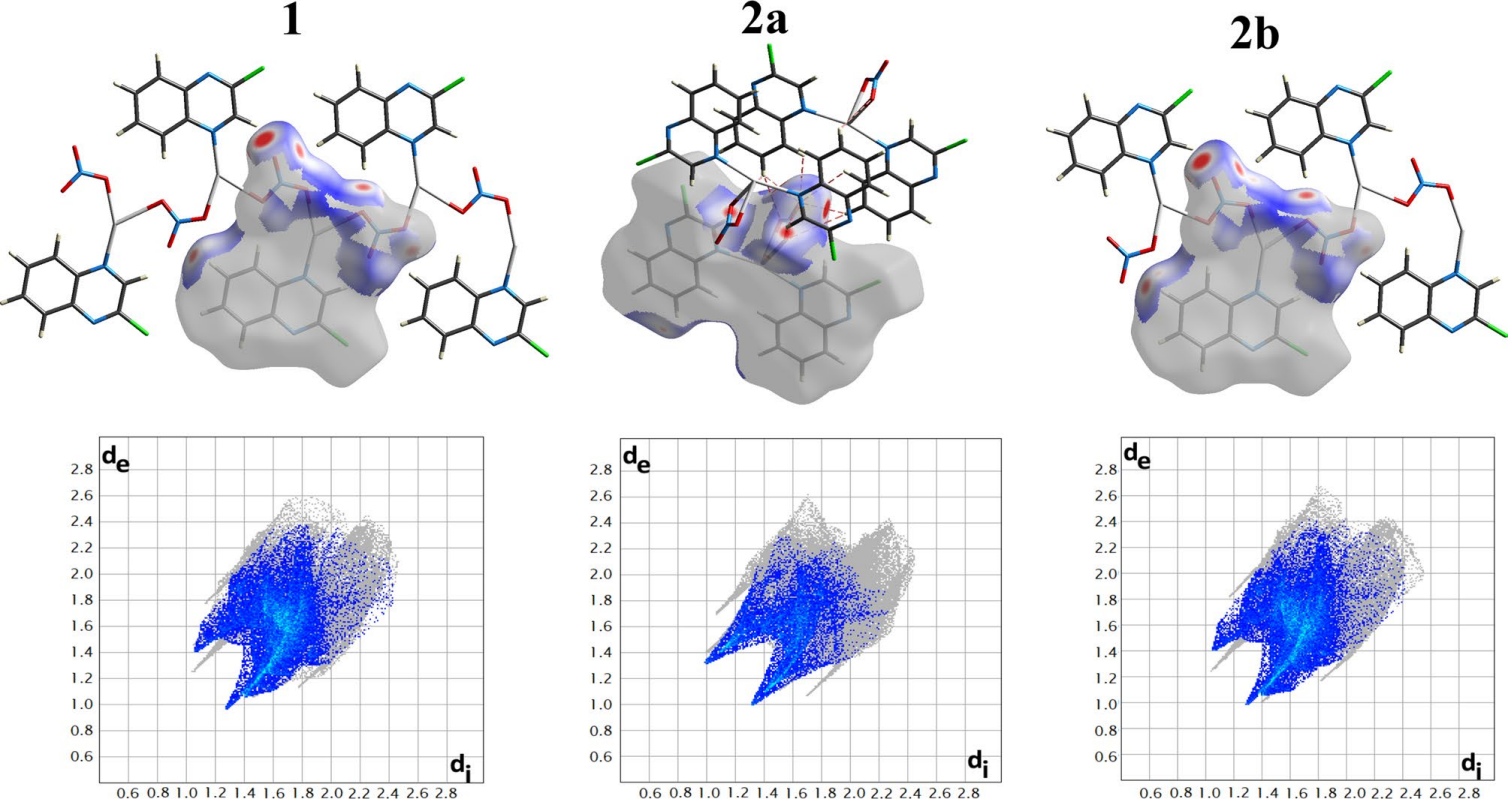
The d_norm_ maps (upper) and fingerprint (lower) plots of the O…H contacts.

The O…H interactions play a significant role in the crystal packing of the three complex units, as shown in Fig. 11. Their percentages to the overall fingerprint plots are 31.3, 20.5, and 31.9% for **1**, **2a**, and **2b**, respectively (Fig. 9). The important role of these interactions in the packing was confirmed by the red spots in the decomposed d_norm_ maps and the sharp spikes pointing to the bottom left corner of the fingerprint plots presented in Fig. 11. These O…H contacts are owing to the interactions that took place between the O-atoms of the nitrate group and the C–H protons from the quinoxaline moiety. It’s worth noting that complexes, **1**, **2a**, and **2b** also have a lot of intermolecular H…Cl interactions which contribute by 14.1, 18.2, and 14.2%, respectively (Fig. 9).

**Figure 12:**
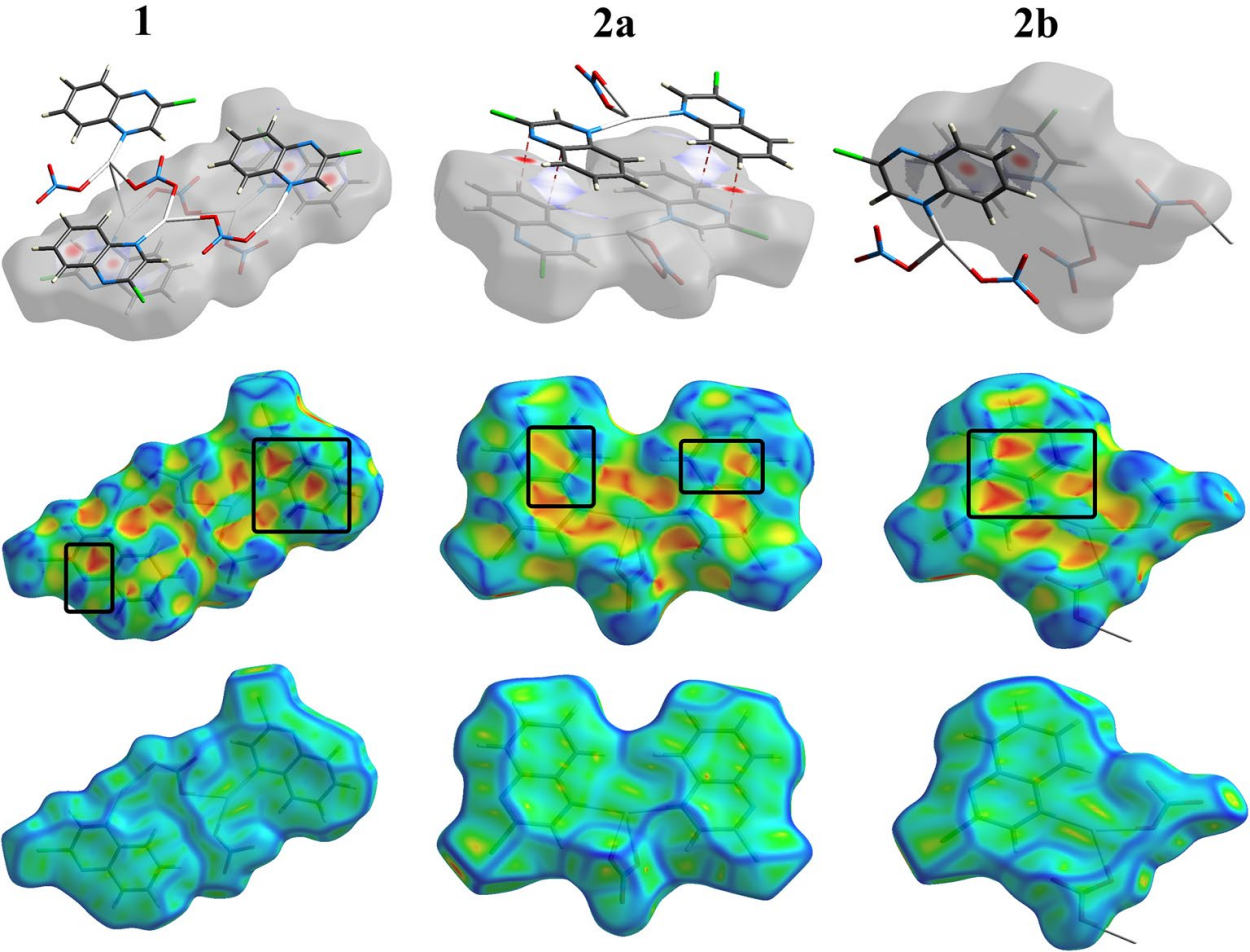
The d_norm_ maps of C…C contact (upper), shape index (middle), and curvedness (lower) of three complexes.

Another important feature of crystal packing is further revealed using Hirshfeld surface analysis which is the π–π stacking interactions. As shown in Fig. 12, all proofs for π–π interactions, including red zones in d_norm_ and red/blue triangles in the shape index with relatively flat areas on the curvedness map, were accomplished. Furthermore, there is a proper amount of C…C and C…N interactions in the molecular structure of the three complex units, as indicated by the fingerprint plots. The contributions of the C…C intermolecular interactions are 7.2, 8.2, and 5.9% of the overall fingerprint plots of **1**, **2a**, and **2b** respectively. Also, the C…N percentages are 5.9, 6.4, and 4.5%, respectively (Fig. 9).

**Figure 13:**
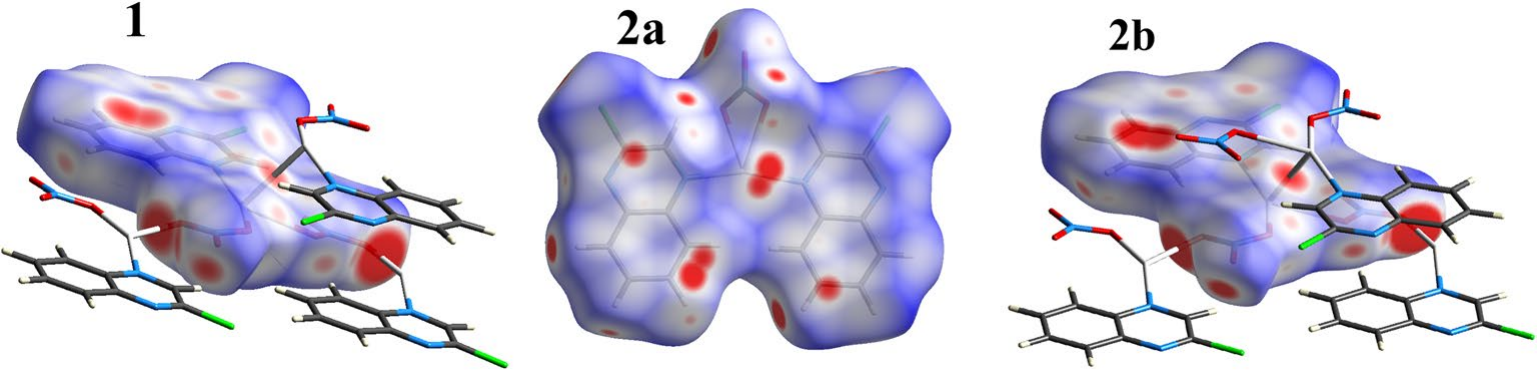
Hirshfeld d_norm_ maps reveal the polymeric nature of **1** and **2b** and the monomeric form of **2a**.

The polymeric nature of **1** and **2b** was clearly identified by the intense red spots neighbouring the Ag atom in the corresponding d_norm_ maps, which are related to the Ag–O and Ag–Ag bonding with the adjacent complex units to construct the 2D coordination polymer, while the molecular compound **2a** is a monomeric complex (Fig. 13).

### Antimicrobial activity

Ag(I) complexes based on various *N*-heterocyclic ligands have been approved to have significant biological properties^11,58^. In this regard, the antimicrobial activities of the studied complexes **1** and **2** were examined. The Minimum Inhibition Concentrations (MIC) of these complexes against MDR microbes were determined, and the results were compared with the activities of some commercial antibiotics. The bacteria used in this study are Gram-negative bacteria (*Proteus morganii, Escherichia coli*, and *Serratia marcescens*) and Gram-positive bacteria (*Bacillus cereus, Micrococcus luteu*, and *Staphylococcus aureus*). The results are presented in Table 1 and displayed graphically in Fig. S9 (Supplementary data).

**Table 1:** Minimum inhibitory concentration (MIC) in µg/mL for complexes **1** and **2**, compared to **2Cl-quinox**, AgNO_3_ and some reference antibiotic drugs.

Antibiotic	Gram positive bacteria			Gram negative bacteria			Fungi	
	S. aureus	M. luteus	B. cereus	E. coli	P. morganii	S. marcescens	A. fumigatus	C. albicans
Amikacin	64	8	16	32	128	128	–	–
Gentamicin	32	16	32	16	96	64	–	–
Streptomycin	128	64	24	32	64	48	–	–
Amoxicillin	32	24	12	192	256	128	–	–
Ampicillin	16	16	8	64	256	256	–	–
Cephradinei	64	64	16	128	96	24	–	–
Cefuroximeii	32	32	24	64	64	16	–	–
Cefoperazoneiii	32	16	16	24	32	32	–	–
Cefepimeiv	12	8	8	32	24	128	–	–
Imipenem	16	32	12	16	256	32	–	–
Meropenem	8	16	6	64	128	64	–	–
Azithromycin	16	16	16	32	96	96	–	–
Clarithromycin	8	24	8	24	64	48	–	–
Nalidixic acid i	64	32	24	16	192	64	–	–
Ciprofloxacinii	32	24	8	32	128	32	–	–
Levofloxaciniii	16	16	12	24	48	128	–	–
Vancomycin	32	24	16	32	128	64	–	–
Ketoconazole	–	–	–	–	–	–	156	312
**2Cl-quinox**	40	256	1250	256	256	256	1250	2500–
AgNO_3_	64	32	256	32	64	96	312	128
Complex 1	256	32	32	256	32	40	1250	312
Complex 2	40	24	32	32	8	48	1250	312

(MIC ≥ 256 indicated no activity).

Roman numbers in superscript show the antibiotic’s generation.

In general, it can be seen from Table 1 that the free **2Cl-quinox** ligand is inactive against all tested bacteria (MIC ≥ 256 µg/mL) except *Staphylococcus aureus* (MIC = 40 µg/mL). On the contrary, complexes **1** and **2** outperformed their free ligand, **2Cl-quinox**, against all tested bacteria except *Staphylococcus aureus* and *E. coli*. Interestingly, complex **2** have better activity than AgNO_3_ against all tested bacteria. The same is true for complex **1** except against *Staphylococcus aureus* and *E. coli.* Complex **1** has good antibacterial potential against *Micrococcus luteu, Bacillus cereus, Proteus morganii* (MIC = 32 µg/mL) and *Serratia marcescens* (MIC = 40 µg/mL) which could be comparable to antibiotics such as Levofloxacin^iii^, Cefepime^iv^, Cefoperazone^iii^, Azithromycin, Cefuroxime^ii^, and Gentamicin. Also, complex **2** exhibited excellent and remarkable efficacy towards all tested microbes, with MIC values ranging between 8 and 48 µg/mL. As a result, it has broad-spectrum activity against MDR (multi drug-resistance) bacteria. The most effective function of **2** is against *Proteus morganii*, with a MIC value of 8 μg/mL. As a result, it is superior to the best-performing antibiotics, Cefoperazone^iii^ (MIC = 32 µg/ml) and Cefepime^iv^ (MIC = 24 µg/ml). Also, Table 2 shows the diameters of the inhibition zones resulting from the treatment of these microbes by the studied compounds. They are often consistent with the MIC results, confirming the broad-spectrum activity of both complexes.

**Table 2:** Diameters of inhibition zone (mm) of the tested compounds against different species of microorganisms.

	Gram positive bacteria			Gram negatvie bacteria			Fungi	
	S. aureus	M. luteus	B. cereus	E. coli	P. morganii	S. marcescens	A. fumigatus	C. albicans
2Cl-quinox	13	8	7	10	7	9	NA	NA
AgNO_3_	14	16	13	15	14	13	NA	10
Complex **1**	10	18	19	13	19	17	20	14
Complex **2**	11	14	23	12	24	17	21	15
Gentamicin	25	22	22	29	11	16	–	–
Ketoconazole	–	–	–	–	–	–	21	22

In addition, the investigated complexes have the same antifungal activity against *Candida albicans* as Ketoconazole as a standard antifungal medication (MIC = 312 µg/mL). On the other hand, both complexes have no antifungal action against *Aspergillus fumigatus* at the applied concentration (MIC = 1250 µg/mL). Based on literature, the antimicrobial properties of Ag(I)-complexes of weak metal–ligand bonds, such as those with Ag–N and Ag–O are expected to be exhibit a broader range of antimicrobial activity than those with Ag–S and Ag–P bonds^24–27^. The results presented in this work are found to be in accordance with the literature.

### Cytotoxic activity

The MTT technique was applied to evaluate the in vitro cytotoxic activity of the studied Ag(I) complexes compared to the free ligand and silver nitrate against the two human lung (A-549) and breast (MCF-7) cancer cell lines. The results are also compared with the clinical anticancer drug, *cis*-platin, under the same experimental conditions. The results of the IC_50_ values in μg/mL are listed in Tables S8–S13 and visually presented in Fig. S10 (Supplementary data)**.**

The studied silver(I) complexes demonstrated high levels cytotoxicity over their free ligand against the examined tumour cell lines. Complex **1** has potential activity against both cancerous cell lines but slightly less than *cis*-platin (Table 3). Interestingly, complex **2** has remarkable greater cytotoxic behaviour (IC_50_ = 5.93 ± 0.52 μg/mL) against lung carcinoma (A-549) than *cis*-platin (IC_50_ = 7.5 ± 0.69 μg/mL) and AgNO_3_ (IC_50_ = 14.70 ± 0.53 μg/mL). This confirms that, the antitumor potential of Ag(I) complexes is related to both the ligand and the metal ion^12,20,33^. In addition, complex **2** exhibits moderate activity (IC_50_ = 9.77 ± 0.74 μg/mL) against the MCF-7 cell line in comparison to *cis*-platin (IC_50_ = 4.59 ± 0.53 μg/mL) and AgNO_3_ (IC_50_ = 2.81 ± 0.97 μg/mL). Hence, AgNO_3_ has the most anticancer effect against the MCF-7 cell line. As we note from Table 3, the cytotoxicity of complex **2** towards both cancerous cell lines is greater than that of complex **1**. This might be attributed to the higher content of silver in complex **2** than in **1**. Similar to the previously reported Ag(I) complexes^12,33^ **1** and **2** showed higher activity towards lung carcinoma (A-549) than breast carcinoma (MCF-7) (Table 3).

**Table 3:** IC_50_ values (in µg/mL) for the Ag(I) complexes, **2Cl-quinox**, AgNO_3_ and *cis*-platin against selected cancer cell lines.

Compound	A-549	MCF-7
Complex **1**	9.11 ± 0.96	17.41 ± 1.83
Complex **2**	5.93 ± 0.52	9.77 ± 0.74
**2Cl-quinox**	143.14 ± 9.78	205.04 ± 11.92
AgNO_3_	14.70 ± 0.53	2.81 ± 0.97
Cis-platin	7.5 ± 0.69	4.59 ± 0.53

## Conclusion

Two novel Ag(I) complexes were self-assembled from the reaction of 2-chloroquinoxaline and silver nitrate in one pot. The single crystal structure of complex **1** comprised a 2D polymer of **[Ag(2Cl-quinox)(NO**_**3**_**)]**_**n**_ sheets via Ag–O and Ag–Ag bondings, while the crystal structure of complex **2** comprised alternating arrays of the **[Ag(2Cl-quinox)(NO**_**3**_**)]**_**n**_ 2D polymer and the **[Ag(2Cl-quinox)**_**2**_**(NO**_**3**_**)]** monomer. Unusually, the 2-chloroquinoxaline ligand in the studied complexes acts as a terminal monodentate ligand and is not involved in the infrastructure of the 2D coordination polymer. Thermogravimetric analysis, FTIR, and NMR spectra are consistent with the complexes’ structures. Both complexes demonstrated broad-spectrum activity against multi drug-resistance (MDR) bacteria. Moreover, the antibacterial potency of complex **2** with a MIC value of 8 μg/mL is higher than that of reference drugs, Cefepime^iv^ and Cefoperazone^iii^ and AgNO_3_ in case of *Proteus morganii*. The cytotoxicity of complex **2** towards lung carcinoma A-549 and breast carcinoma MCF-7 cell lines is greater than that of complex **1**. This might be attributed to the higher silver content in complex **2** than complex **1**.

## Supplementary Information

Supplementary Information.

## Data Availability

All data generated or analyzed during this study are included in this published article [and its supplementary information files].
