# Supplementary material for: One pot synthesis of two potent Ag(I) complexes with quinoxaline ligand, X-ray structure, Hirshfeld analysis, antimicrobial, and antitumor investigations
Source: Sci Rep. 2022 Dec 3;12:20881. doi: 10.1038/s41598-022-24030-x (PMC9719528; doi:10.1038/s41598-022-24030-x)
Supplement: Supplementary file 1 — Supplementary Information. [file 41598_2022_24030_MOESM1_ESM.docx]

**One pot synthesis of two potent Ag(I) complexes with quinoxaline ligand: X-ray structure, Hirshfeld analysis, antimicrobial, and antitumor investigations.**

Mostafa A. El-Naggar^1^, Mona Mohammed Sharaf ^2^, Jörg H. Albering^3^, Morsy A. M. Abu-Youssef^1*^, Taher S. Kassem^1^, Saied M. Soliman^1*^, Ahmed M. A. Badr^1^

^1^ Department of Chemistry, Faculty of Science, Alexandria University, P.O. Box 426, Ibrahimia, Alexandria 21321, Egypt. Emails: [saied1soliman@yahoo.com](mailto:saied1soliman@yahoo.com) (SMS) and  [morsy5@alexu.edu.eg](mailto:Morsy5@AlexU.edu.eg) (MAMA).

^2^Protein Research Department, Genetic Engineering and Biotechnology Research Institute, City of Scientific Research and Technological Applications, Alexandria, Egypt

^3^ Graz University of Technology, Mandellstr. 11/III, A-8010 Graz, Austria

**
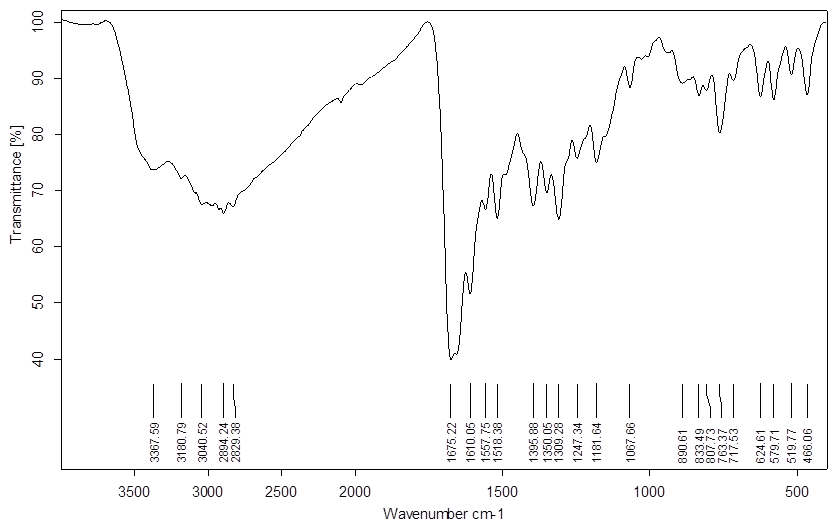
**

**Fig. S1** FTIR spectrum of the free ligand (**2Cl-quinox**)


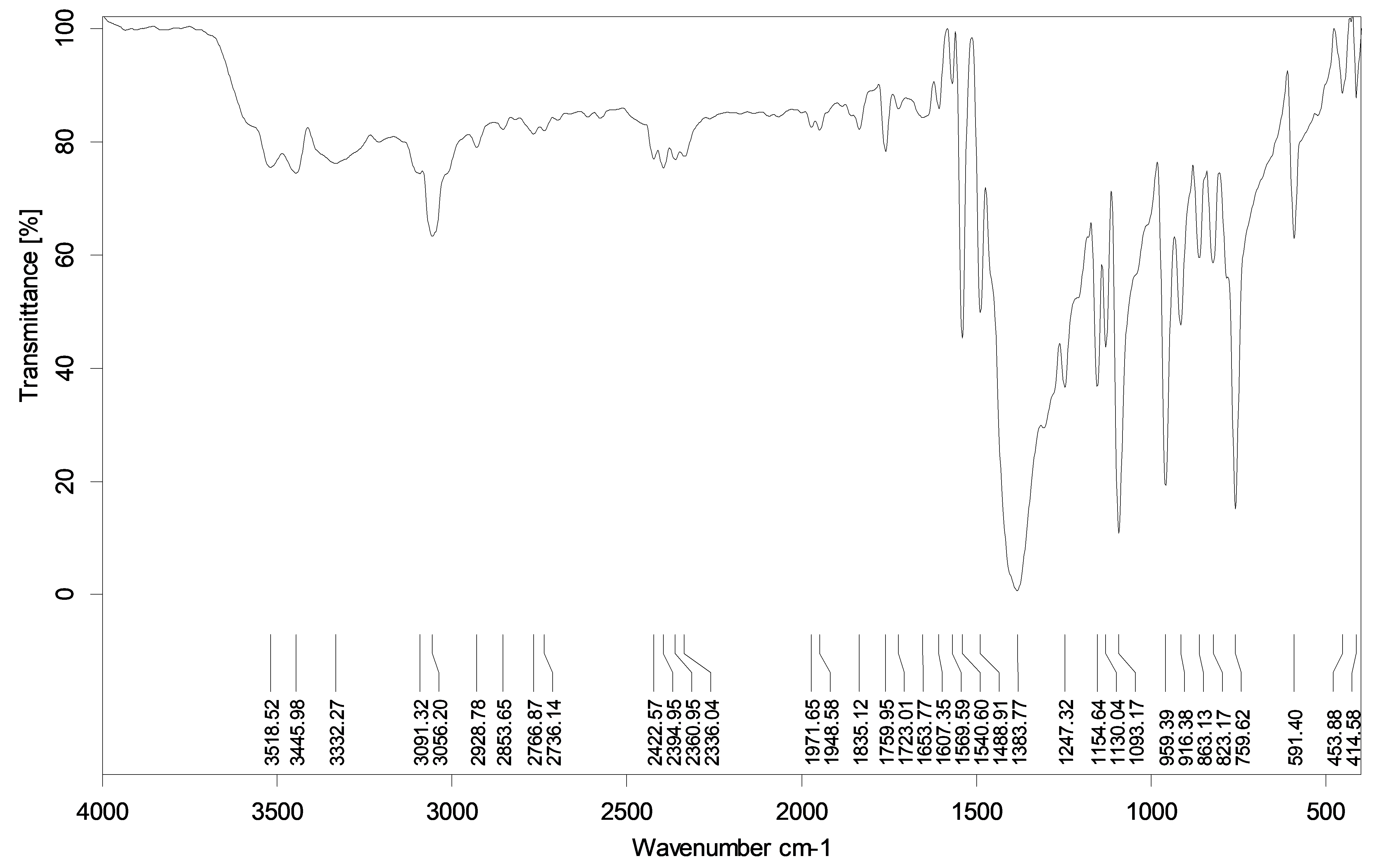


**Fig. S2** FTIR spectrum of the complex **1**; **[Ag(2Cl-quinox)(NO_3_)]**.

**
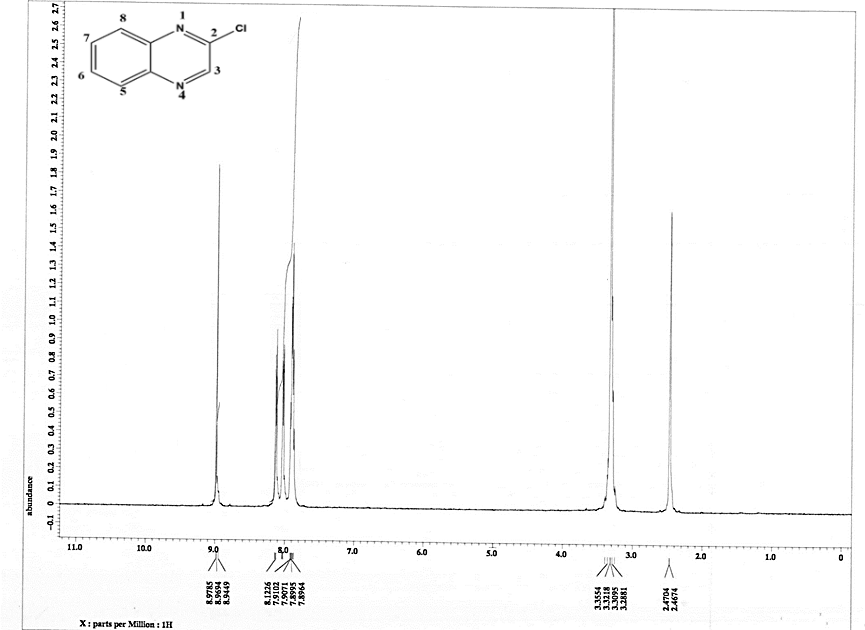
Fig. S3** ^1^H NMR spectrum of the free ligand **2Cl-quinox**.

**
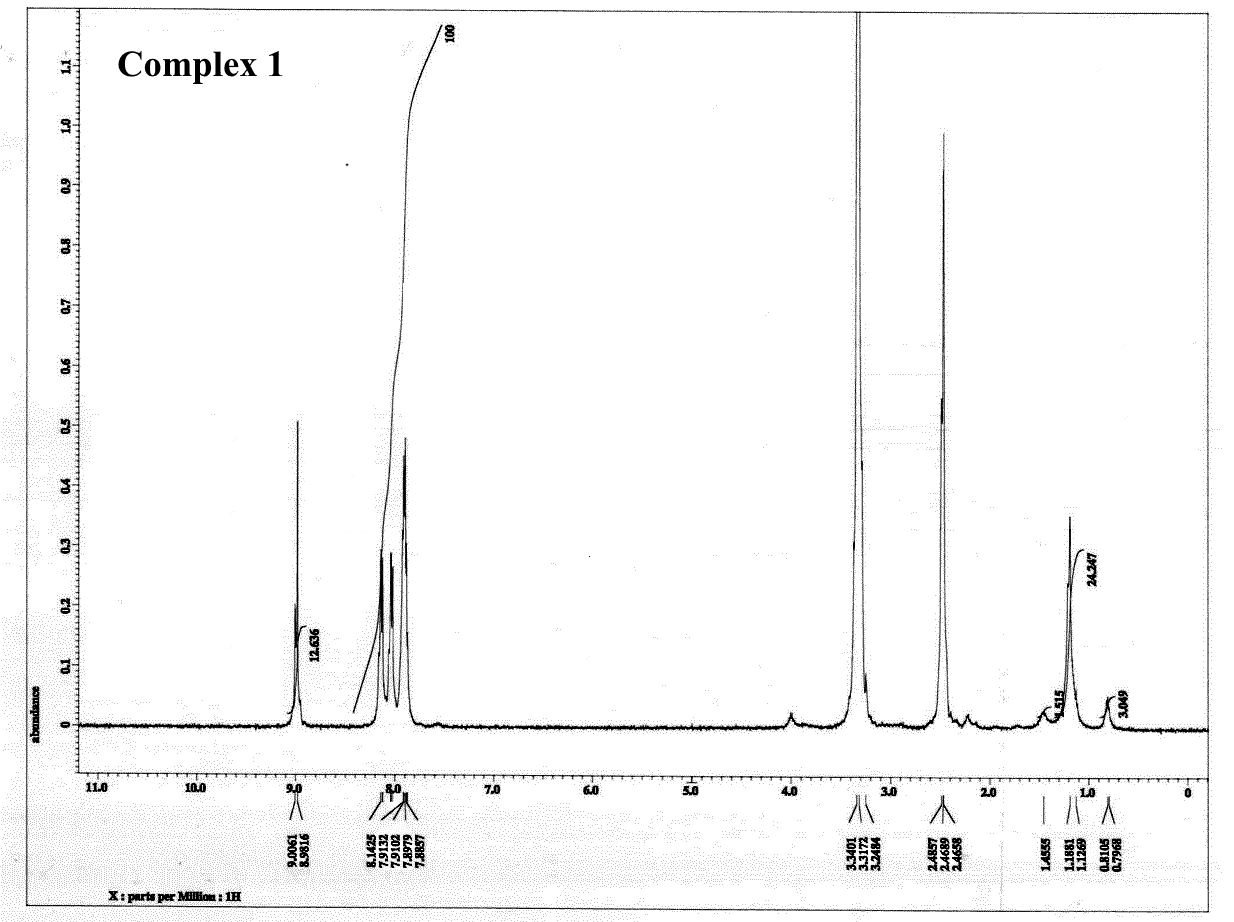
Fig. S4** ^1^H NMR spectrum of complex **1**.


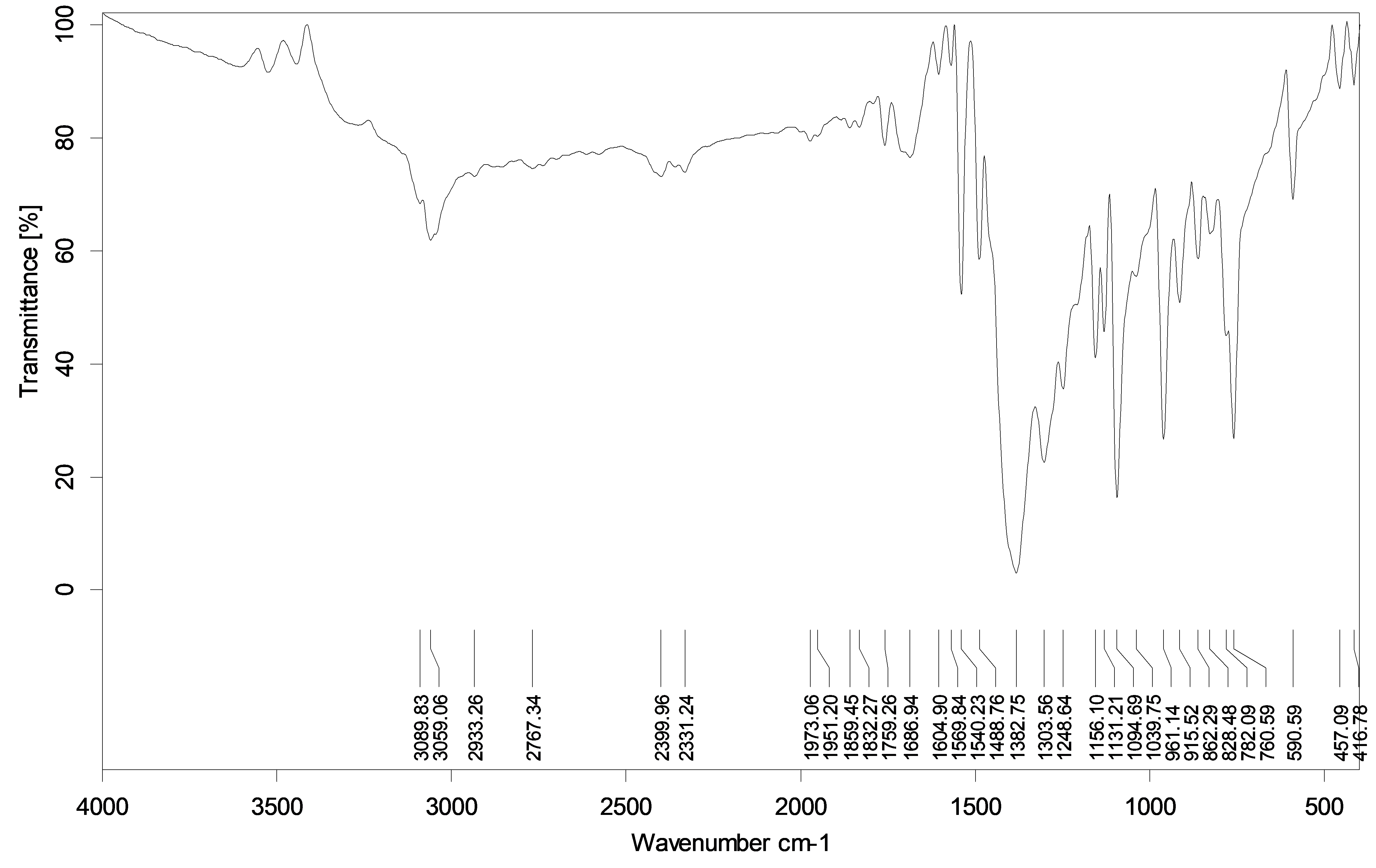


**Fig. S5** FTIR spectrum of the complex **2**; **[Ag_3_(2Cl-quinox)_4_(NO_3_)_3_]**.

**Fig. S6** ^1^H NMR spectrum of complex **2**
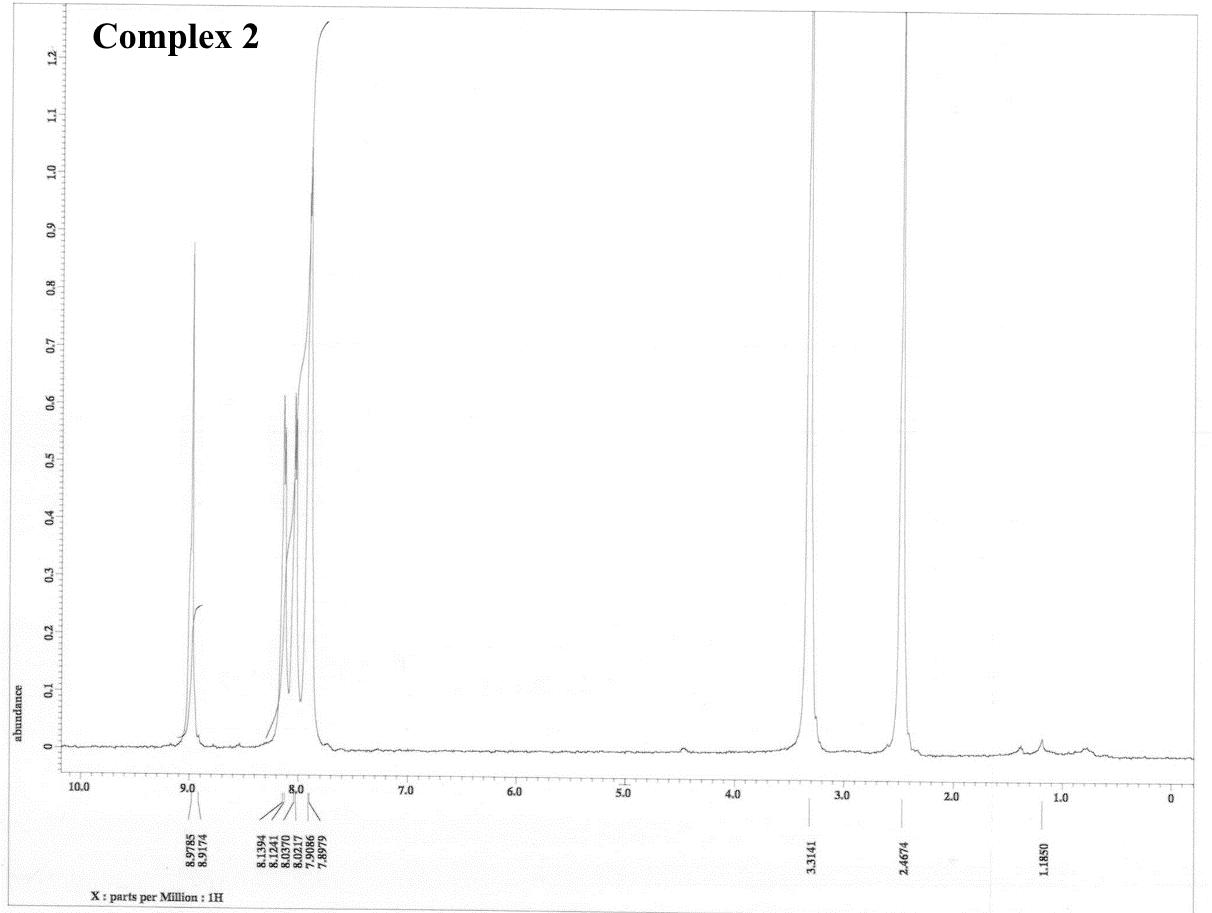
.


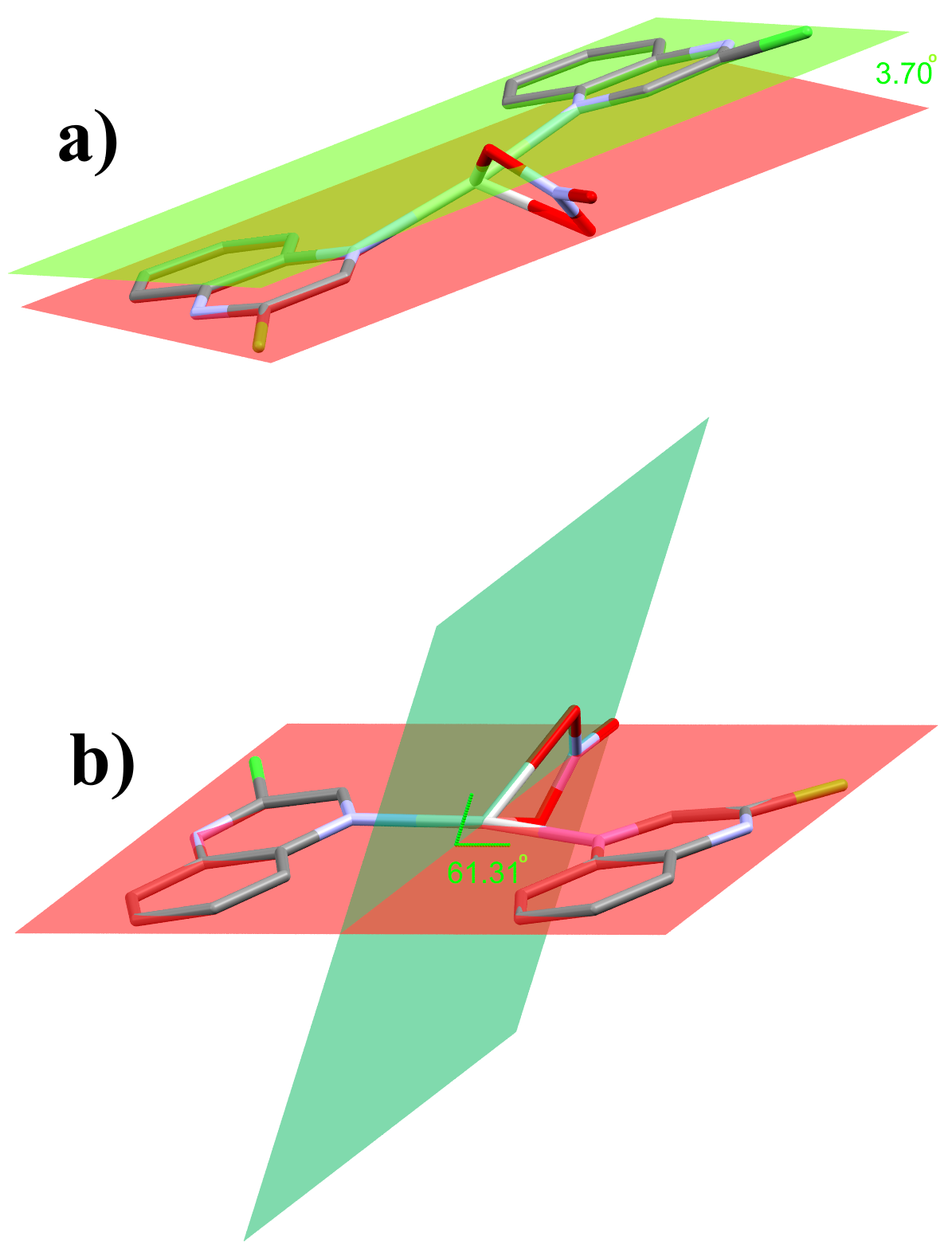


**Fig. S7.** The twist angle between the planes of the two quinoxaline units (Part **a**) and the angle between the mean planes of the quinoxaline moieties and the bidentate nitrate group (Part **b**).

**
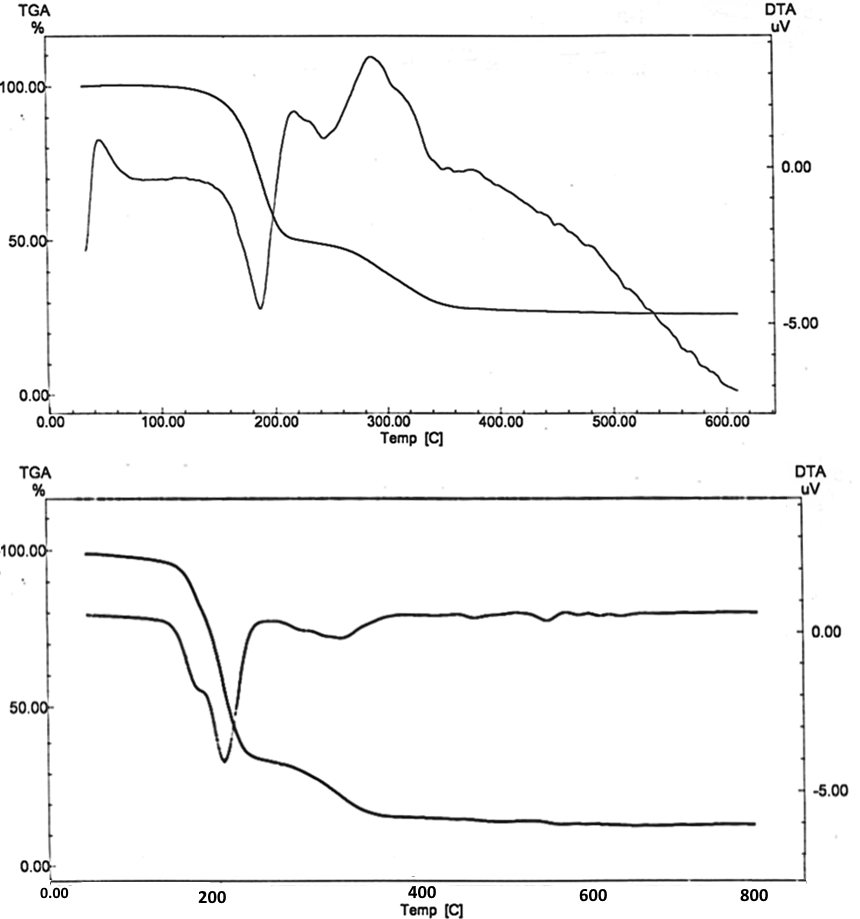
Fig. S8.** TG and DTA curves of complex **1** (upper) and complex **2** (lower).

**
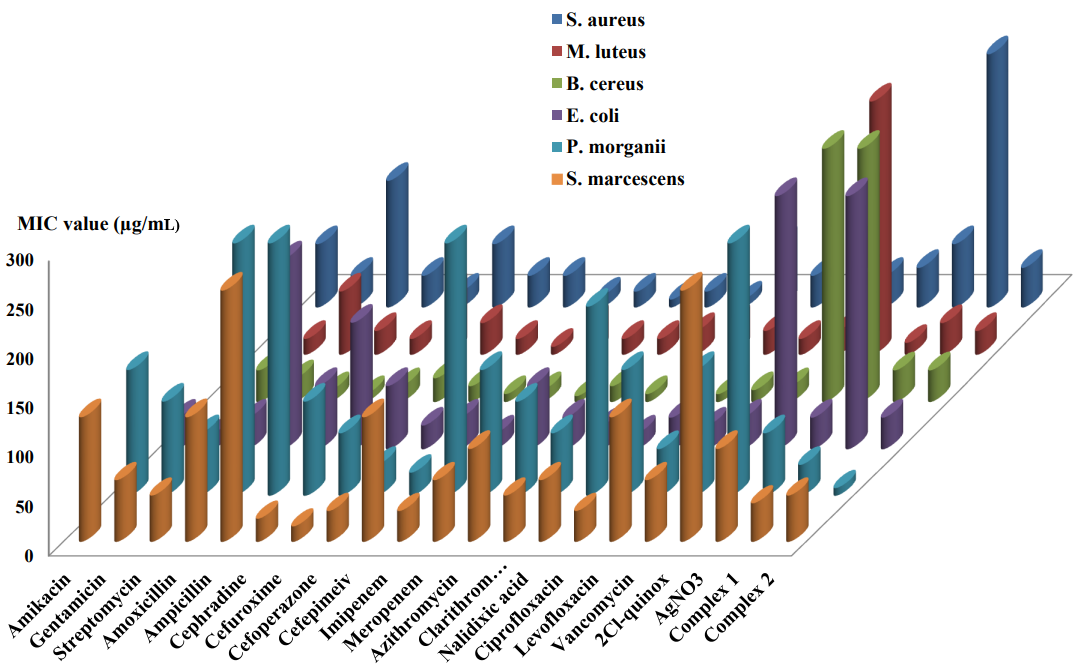
**

**Fig. S9** Summary of the antibacterial study displaying Minimum Inhibitory Concentrations (MIC) in μg/mL, thus lowest values corresponding to the most potent compounds.


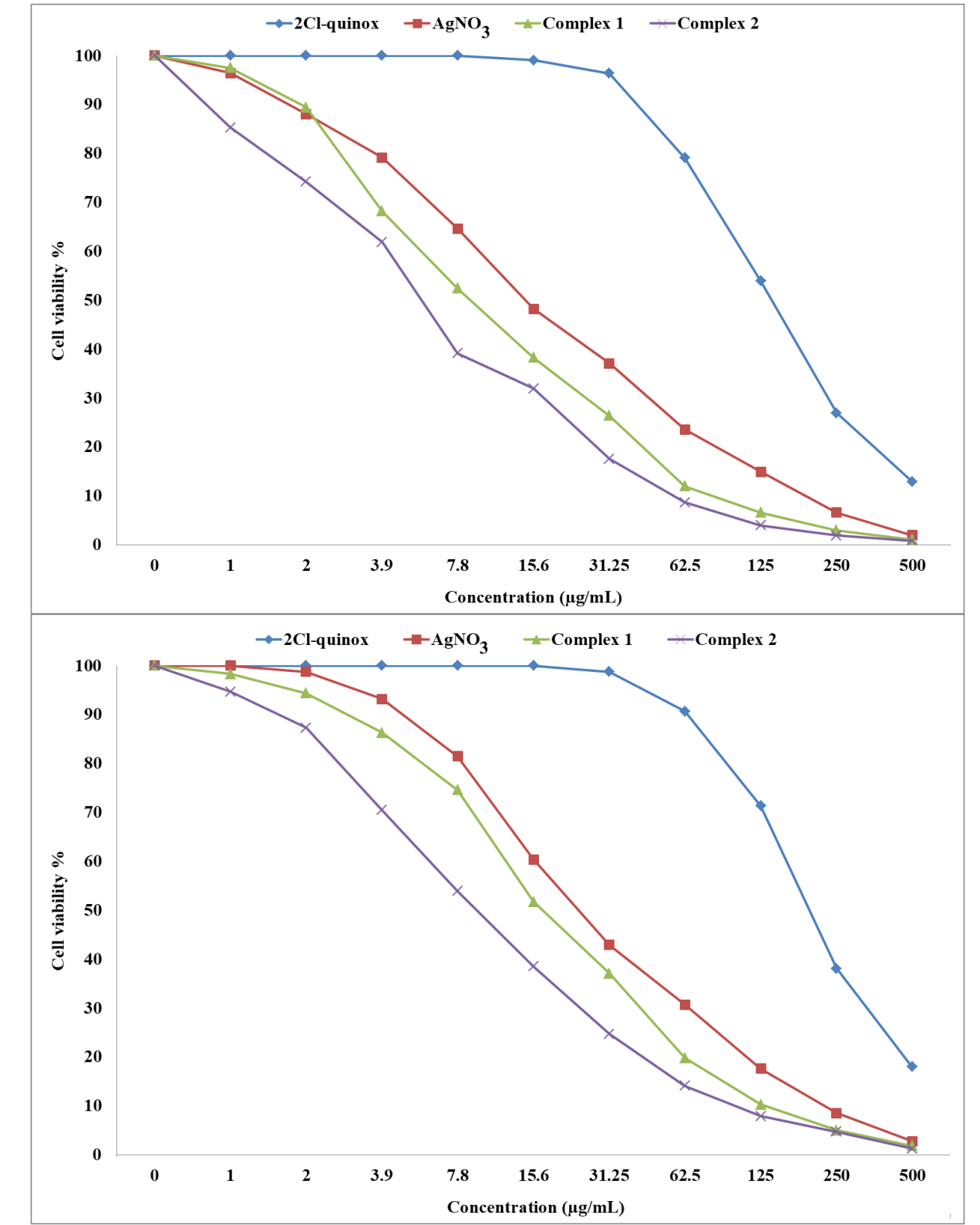


**Fig. S10.** The anticancer activity of the studied complexes, **2Cl-quinox** and AgNO_3_ against A-549 lung (upper) and MCF-7 breast (lower) carcinoma cells.

**Table S1.** Crystallographic details and Crystal refinement parameters for **1** and **2**.

| Compound number | **1** | **2** |
| --- | --- | --- |
| Empirical formula | C_8_H_5_AgClN_3_O_3_ | C_32_H_20_Ag_3_Cl_4_N_11_O_9_ |
| Formula weight | 334.47 g/mol | 1168.00 g/mol |
| Temperature (K) | 100(2) | 100(2) |
| Wavelength (Å) | 0.71073 | 0.71073 |
| Crystal system | Monoclinic | Monoclinic |
| Space group | C2/c | C2/c |
| Unit cell dimensions | a = 27.638(3) Å | a = 50.235(2) Å |
|  | b = 9.6496(11) Å | b = 9.7041(4) Å |
|  | c = 7.2976(8) Å | c = 7.3220(3) Å |
|  | α = 90° | α = 90° |
|  | β = 100.438(2)° | β = 91.536(2)° |
|  | γ = 90° | γ = 90° |
| Volume (Å^3^) | 1914.0(4) | 3568.1(3) |
| Z | 8 | 4 |
| Density (calculated) | 2.321 g/cm^3^ | 2.174 g/cm^3^ |
| Absorption coefficient | 2.378 mm^-1^ | 2.005 mm^-1^ |
| F(000) | 1296 | 2280 |
| Crystal size (mm^3^) | 0.12 x 0.27 x 0.42 | 0.04 x 0.12 x 0.54 |
| Theta range for data collection | 1.50 to 30.00° | 2.14 to 27.50° |
| Index ranges | -36≤h≤38, | -65≤h≤64, |
|  | -13≤k≤13, | -11≤k≤12, |
|  | -10≤l≤10 | -9≤l≤5 |
| Reflections collected | 10576 | 14823 |
| Independent reflections | 2795 [R(int) = 0.0209] | 4080 [R(int) = 0.0290] |
| Completeness to theta = | 99.9% | 99.4% |
| Absorption correction | Multiscan | Multiscan |
| Max. and min. transmission | 0.7630 and 0.4350 | 0.9300 and 0.4140 |
| Refinement method | Full-matrix least-squares on F^2^ | Full-matrix least-squares on F^2^ |
| Data / restraints / parameters | 2795 / 0 / 145 | 4080 / 0 / 268 |
| Goodness-of-fit on F^2^ | 1.158 | 1.024 |
| Final R indices [I>2sigma(I)] | R1 = 0.0213, wR2 = 0.0509 | R1 = 0.0277, wR2 = 0.0675 |
| R indices (all data) | R1 = 0.0219, wR2 = 0.0512 | R1 = 0.0349, wR2 = 0.0710 |
| Largest diff. peak and hole | 0.855 and -0.815 e.Å^-3^ | 2.161 and -0.442 e.Å^-3^ |
| **CCDC** | 2204505 | 2204506 |

**Table S2.** Selected bond lengths [Å] and angles [°] for **1**.

| **Bond length** |  |
| --- | --- |
| Ag(1)-N(1) | 2.2286(15) |
| Ag(1)-O(1) | 2.5502(15) |
| Ag(1)-O(2)#1 | 2.2602(14) |
| Ag(1)-Ag(1)#2 | 3.1008(4) |
| **Bond angles** |  |
| N(1)-Ag(1)-O(1) | 130.21(5) |
| N(1)-Ag(1)-O(2)#1 | 153.57(6) |
| O(1)-Ag(1)-O(2)#1 | 74.65(5) |
| N(1)-Ag(1)-Ag(1)#2 | 104.68(4) |
| O(2)-Ag(1)-Ag(1)#2 | 74.37(4) |
| O(1)-Ag(1)-Ag1#2 | 67.58(4) |

Symmetry codes: #1 1/2-x,1/2+y,2.5-z #2 1/2-x,1/2-y,2-z

**Table S3.** Hydrogen bonds for complex **1** [Å and °].

| **D-H...A** | **d(D-H)** | **d(H...A)** | **d(D...A)** | **<(DHA)** |
| --- | --- | --- | --- | --- |
| C(1)-H(1)...O(3) | 0.95 | 2.31 | 3.072(2) | 137 |
| C(5)-H(5)...N(2)#1 | 0.95 | 2.61 | 3.544(2) | 169 |
| C(7)-H(7)...O(3) #2 | 0.95 | 2.56 | 3.446(2) | 156 |
| C(8)-H(8)...O(1)#3 | 0.95 | 2.55 | 3.467(2) | 162 |

Symmetry codes: #1 -x,1-y,1-z #2 x,1-y,-1/2+z #3 1/2-x,1/2+y,2.5-z

**Table S4.** Selected bond lengths [A] and angles [°] for **2**

| **Bond length** |  |
| --- | --- |
| Ag(1)-N(1) | 2.243(2) |
| Ag(1)-O(1) | 2.613(2) |
| Ag(2)-N(4) | 2.231(2) |
| Ag(2)-O(3)#2 | 2.2588(18) |
| Ag(2)-O(4) | 2.548(2) |
| Ag(2)-Ag(2)#3 | 3.1100(4) |
| **Bond angles** |  |
| N(1)-Ag(1)-N(1)#1 | 162.73(12) |
| N(4)-Ag(2)-O(3)#2 | 152.15(8) |
| N(4)-Ag(2)-O(4) | 130.38(7) |
| O(3)#2-Ag(2)-O(4) | 75.86(6) |
| N(4)-Ag(2)-Ag(2)#3 | 104.47(5) |
| O(3)#2-Ag(2)-Ag(2)#3 | 75.25(5) |
| O(4)-Ag(2)-Ag(2)#3 | 66.79(5) |

Symmetry codes: #1 -x,y,1/2-z #2 1/2-x,-1/2+y,-1/2-z #3 1/2-x,1/2-y,-z

**Table S5.** Hydrogen bonds for complex **2** [Å and °]

| **D-H...A** | **d(D-H)** | **d(H...A)** | **d(D...A)** | **<(DHA)** |
| --- | --- | --- | --- | --- |
| C(1)-H(1)...O(1) | 0.95 | 2.55 | 3.285(3) | 134 |
| C(4)-H(4)...N(5) | 0.95 | 2.50 | 3.441(3) | 170 |
| C(6)-H(6)...O(2)#1 | 0.95 | 2.38 | 3.146(3) | 138 |
| C(12)-H(12)...N(2) | 0.95 | 2.71 | 3.654(3) | 173 |

Symmetry code: #1 x,-1+y,z

**Table S6.**  ^1^H NMR analysis data for Ag(I) complexes and their free ligand.

| **Fragment** | **C(3)H** | **C(5)H** | **C(6)H & C(7)H** | **C(8)H** |
| --- | --- | --- | --- | --- |
| **2Cl-quinox** | 8.97 | 8.03 | 7.89 | 8.12 |
| **Complex 1** | 8.99 | 8.03 | 7.89 | 8.12 |
| **Complex 2** | 8.94 | 8.03 | 7.90 | 8.12 |

**Table S7** Thermoanalytical data for complexes **1** and **2**.

| **Complex** | **Step** | **TG range ˚C** | **Exp. Loss %** | **Theor. Loss %** | **Fragment**  **released** | **Type** | **Residue** |
| --- | --- | --- | --- | --- | --- | --- | --- |
| **1** | 1^st^ | 38-220^°^C | 49.639 | 49.211 | 2Cl-quinox | Endo. | [NO_2_+ 0.5O_2_]+ Ag |
|  | 2^nd^ | 220-600^°^C | 18.175 | 18.539 | [NO_2_ + 0.5O_2_] | Exo. | Metallic Silver [Ag] |
| **2** | 1^st^ | 50-240˚C | 55.423 | 56.367 | 4 2Cl-quinox | Endo. | 3[NO_2_ + 0.5O_2_] + 3Ag |
|  | 2^nd^ | 240-800˚C | 15.638 | 15.926 | 3 [NO_2_ + 0.5O_2_] | Exo. | Metallic Silver 3[Ag] |

**Table S8** Evaluation of cytotoxicity against A-549 cell line for 2Cl-quinox.

| Sample conc. (µg/ml) | Viability % | Inhibitory % | S.D. (±) |
| --- | --- | --- | --- |
| 500 | 12.87 | 87.13 | 1.25 |
| 250 | 26.91 | 73.09 | 2.83 |
| 125 | 53.89 | 46.11 | 2.17 |
| 62.5 | 79.04 | 20.96 | 1.62 |
| 31.25 | 96.32 | 3.68 | 0.84 |
| 15.6 | 99.06 | 0.94 | 0.72 |
| 7.8 | 100 | 0 |  |
| 3.9 | 100 | 0 |  |
| 2 | 100 | 0 |  |
| 1 | 100 | 0 |  |
| 0 | 100 | 0 | 0 |

*IC_50 =_ 143.14 ± 9.78 µg/ml.*

**Table S9** Evaluation of cytotoxicity against A-549 cell line for 1.

| Sample conc. (µg/ml) | Viability % | Inhibitory % | S.D. (±) |
| --- | --- | --- | --- |
| 500 | 1.04 | 98.96 | 0.32 |
| 250 | 2.92 | 97.08 | 0.16 |
| 125 | 6.58 | 93.42 | 0.31 |
| 62.5 | 11.96 | 88.04 | 0.72 |
| 31.25 | 26.34 | 73.66 | 0.68 |
| 15.6 | 38.26 | 61.74 | 1.78 |
| 7.8 | 52.37 | 47.63 | 1.95 |
| 3.9 | 68.15 | 31.85 | 1.31 |
| 2 | 89.40 | 10.6 | 0.68 |
| 1 | 97.46 | 2.54 | 0.22 |
| 0 | 100 | 0 | 0 |

*IC_50 =_ 9.11 ± 0.96 µg/ml.*

**Table S10** Evaluation of cytotoxicity against A-549 cell line for 2.

| Sample conc. (µg/ml) | Viability % | Inhibitory % | S.D. (±) |
| --- | --- | --- | --- |
| 500 | 0.79 | 99.21 | 0.23 |
| 250 | 1.87 | 98.13 | 0.09 |
| 125 | 3.94 | 96.06 | 0.18 |
| 62.5 | 8.62 | 91.38 | 0.24 |
| 31.25 | 17.48 | 82.52 | 0.66 |
| 15.6 | 31.94 | 68.06 | 1.08 |
| 7.8 | 39.08 | 60.92 | 2.46 |
| 3.9 | 61.87 | 38.13 | 2.39 |
| 2 | 74.16 | 25.84 | 0.48 |
| 1 | 85.23 | 14.77 | 0.11 |
| 0 | 100 | 0 | 0 |

*IC_50 =_ 5.93 ± 0.52 µg/ml.*

**Table S11** Evaluation of cytotoxicity against MCF-7 cell line for 2Cl-quinox.

| Sample conc. (µg/ml) | Viability % | Inhibitory % | S.D. (±) |
| --- | --- | --- | --- |
| 500 | 17.92 | 82.08 | 1.86 |
| 250 | 38.04 | 61.96 | 2.32 |
| 125 | 71.27 | 28.73 | 3.15 |
| 62.5 | 90.63 | 9.37 | 1.51 |
| 31.25 | 98.74 | 1.26 | 0.62 |
| 15.6 | 100 | 0 |  |
| 7.8 | 100 | 0 |  |
| 3.9 | 100 | 0 |  |
| 2 | 100 | 0 |  |
| 1 | 100 | 0 |  |
| 0 | 100 | 0 | 0 |

*IC_50 =_ 205.04 ± 11.92 µg/ml.*

**Table S12** Evaluation of cytotoxicity against MCF-7 cell line for 1.

| Sample conc. (µg/ml) | Viability % | Inhibitory % | S.D. (±) |
| --- | --- | --- | --- |
| 500 | 1.79 | 98.21 | 0.37 |
| 250 | 4.97 | 95.03 | 0.29 |
| 125 | 10.23 | 89.77 | 0.61 |
| 62.5 | 19.74 | 80.26 | 0.52 |
| 31.25 | 37.02 | 62.98 | 1.46 |
| 15.6 | 51.70 | 48.3 | 2.31 |
| 7.8 | 74.56 | 25.44 | 1.52 |
| 3.9 | 86.29 | 13.71 | 0.73 |
| 2 | 94.31 | 5.69 | 0.65 |
| 1 | 98.28 | 1.72 | 0.48 |
| 0 | 100 | 0 | 0 |

*IC_50 =_ 17.41 ± 1.83 µg/ml.*

**Table S13** Evaluation of cytotoxicity against MCF-7 cell line for 2.

| Sample conc. (µg/ml) | Viability % | Inhibitory % | S.D. (±) |
| --- | --- | --- | --- |
| 500 | 1.23 | 98.77 | 0.41 |
| 250 | 4.65 | 95.35 | 0.39 |
| 125 | 7.89 | 92.11 | 0.23 |
| 62.5 | 14.06 | 85.94 | 0.12 |
| 31.25 | 24.63 | 75.37 | 0.59 |
| 15.6 | 38.46 | 61.54 | 1.62 |
| 7.8 | 53.91 | 46.09 | 2.73 |
| 3.9 | 70.42 | 29.58 | 1.64 |
| 2 | 87.29 | 12.71 | 0.93 |
| 1 | 94.67 | 5.33 | 0.45 |
| 0 | 100 | 0 | 0 |

*IC_50 =_ 9.77 ± 0.74 µg/ml.*

**Method S1**

**Testing of Antimicrobial Activity**

“The antimicrobial activities of the studied Ag(I) complexes were determined according to the recommendations of NCCLS4038 by the use of the broth microdilution method. Minimum inhibitory concentrations (MICs) for the tested compounds were conducted using *S. aureus, M. luteus* and *B. cereus* as Gram-positive bacteria*; E. coli, P. morganii and S. marcescens* as Gram negative bacteria, and two yeasts*, A. fumigatus and C. albicans*. The tested compounds were dissolved in DMSO to give a stock solution that was subsequently diluted in the growth medium to give 1.5 serial dilutions from 256–0.5 μg/mL medium. To ensure full solubility of the tested materials, 5% DMSO was present in all bioassay media, a concentration which had no antibacterial effect on its own. Bacteria were cultured in Mueller Hinton Broth (MHB) for 24 h at 35 ˚C with 105 CFU/mL culture filtrate. MIC values correspond to the lowest concentration that inhibited the bacterial growth.

In case of inhibition zone determination, 100 μl of the test bacteria/fungi were grown in 10 mL of fresh media until they reached a count of approximately 108 cells/ml for bacteria or 105 cells/mL for fungi. One hundred μl of microbial suspension was spread onto agar plates corresponding to the broth in which they were maintained and tested for susceptibility by well diffusion method. One hundred µL of each sample (at 10 mg/ml) was added to each well (6 mm diameter holes cut in the agar gel). The plates were incubated for 24-48 h at 37 °C (for bacteria and yeast) and for 48 h at 28 °C (for filamentous fungi). After incubation, the microorganism's growth was observed. The resulting inhibition zone diameters were measured in millimeters and used as criterion for the antimicrobial activity. If an organism is placed on the agar it will not grow in the area around the well if it is susceptible to the chemical. This area of no growth around the disc is known as a "Zone of inhibition" or "Clear zone". The size of the clear zone is proportional to the inhibitory action of the compound under investigation. Solvent controls (DMSO) were included in every experiment as negative controls. DMSO was used for dissolving the tested compounds and showed no inhibition zones, confirming that it has no influence on growth of the tested microorganisms. Positive controls were also performed using gentamycin as standard antibacterial drugs and ketoconazole as standard antifungal drug.

**Method S2**

Evaluation of Cytotoxic Effects against the two human lung (A-549) and breast (MCF-7) cancer cell lines.

Mammalian cell line A-549 (human Lung Carcinoma) and MCF-7 (human Breast Carcinoma) cells were obtained from the American Type Culture Collection (ATCC, Rockville, MD). Dimethyl sulfoxide (DMSO), MTT and trypan blue dye was purchased from Sigma (St. Louis, Mo., USA). Fetal Bovine serum, RPMI-1640, HEPES buffer solution, L-glutamine, gentamycin and 0.25% Trypsin-EDTA were purchased from Lonza (Belgium).

**Cell line Propagation:**

The cells were grown on RPMI-1640 medium supplemented with 10% inactivated fetal calf serum and 50µg/mL Gentamycin. The cells were maintained at 37ºC in a humidified atmosphere with 5% CO_2_ and were subcultured two to three times a week.

**Cytotoxicity evaluation using viability assay:**

For antitumor assays, the tumor cell lines were suspended in medium at concentration 5x10^4^ cell/well in Corning® 96-well tissue culture plates, then incubated for 24 hr. The tested compounds were then added into 96-well plates (three replicates) to achieve twelve concentrations for each compound. Six vehicle controls with media or 0.5 % DMSO were run for each 96 well plate as a control. After incubating for 24 h, the numbers of viable cells were determined by the MTT test. Briefly, the media was removed from the 96 well plates and replaced with 100 µL of fresh culture RPMI 1640 medium without phenol red then 10 µL of the 12 mM MTT stock solution (5 mg of MTT in 1 mL of PBS) to each well including the untreated controls. The 96 well plates were then incubated at 37°C and 5% CO_2_ for 4 hours. An 85 µL aliquot of the media was removed from the wells, and 50 µL of DMSO was added to each well and mixed thoroughly with the pipette and incubated at 37°C for 10 min. Then, the optical density was measured at 590 nm with the microplate reader (SunRise, TECAN, Inc, USA) to determine the number of viable cells and the percentage of viability was calculated as [(ODt/ODc)]x100% where ODt is the mean optical density of wells treated with the tested sample and ODc is the mean optical density of untreated cells. The relation between surviving cells and drug concentration is plotted to get the survival curve of each tumor cell line after treatment with the specified compound. The 50% inhibitory concentration (IC_50_), the concentration required to cause toxic effects in 50% of intact cells, was estimated from graphic plots of the dose response curve for each conc. using Graphpad Prism software (San Diego, CA. USA).
